# Long‐read sequencing‐based atlas of tissue‐specific expression of *DNM1L* transcript variants

**DOI:** 10.1111/febs.70568

**Published:** 2026-04-29

**Authors:** Feng Yan, Jafar S. Jabbari, Naomi X. L. Ling, Anne M. Kong, Jia Q. Truong, Marnie L. Maddock, Ayeshah A. Rosdah, Christopher G. Langendorf, Lifang Zhang, Jarmon G. Lees, Sebastian Bass‐Stringer, Danise Ann Onda, Alice Pébay, Mirella Dottori, Kim Loh, Jessica K. Holien, Sean Lal, Nadia M. Davidson, Jonathan S. Oakhill, Shiang Y. Lim

**Affiliations:** ^1^ The Walter and Eliza Hall Institute of Medical Research Parkville Australia; ^2^ Department of Medical Biology The University of Melbourne Parkville Australia; ^3^ St Vincent's Institute of Medical Research Fitzroy Australia; ^4^ STEM College, Engineering and Health RMIT University Melbourne Australia; ^5^ School of Medical, Indigenous and Health Sciences, Molecular Horizons University of Wollongong Australia; ^6^ Faculty of Medicine Universitas Sriwijaya Palembang Indonesia; ^7^ Department of Medicine and Surgery University of Melbourne Parkville Australia; ^8^ Drug Discovery Biology, Faculty of Pharmacy and Pharmaceutical Sciences Monash University Parkville Australia; ^9^ Department of Anatomy and Physiology The University of Melbourne Australia; ^10^ CellTellus Laboratory Melbourne Australia; ^11^ Faculty of Health Sciences Australian Catholic University Melbourne Australia; ^12^ Charles Perkins Centre The University of Sydney Australia; ^13^ Department of cardiology Royal Prince Alfred Hospital Camperdown Australia; ^14^ National Heart Research Institute Singapore National Heart Centre Singapore Singapore

**Keywords:** cardiomyocytes, *DNM1L*, Drp1, isoforms, left ventricle, mitochondrial dynamics

## Abstract

Dynamin‐related protein 1 (Drp1), encoded by *DNM1L*, is essential for mitochondrial fission, but its functional roles remain unclear due to isoform‐specific effects from alternative splicing. Short‐read RNA sequencing fails to resolve full‐length isoforms involving distant exons, limiting our understanding. Here, we applied targeted long‐read sequencing to profile full‐length *DNM1L* transcripts in human left ventricle and induced pluripotent stem cell‐derived cardiomyocytes, recovering all annotated isoforms with conserved expression patterns and isoforms 1–4 being the most abundant. Functional assays revealed that isoform abundance does not predict enzymatic activity. Extending this to six different mouse tissues, we identified distinct, tissue‐enriched expression profiles. Functional rescue in Drp1‐knockout mouse embryonic fibroblasts showed isoform‐dependent differences in mitochondrial fission. Isoforms lacking the A‐insert robustly rescued mitochondrial fission, with maximal activity observed for variants also lacking the B‐insert (e.g. isoform b), consistent with a modulatory role of exon 3 in Drp1 activity. Our cross‐species atlas integrates long‐read transcriptomics with functional validation, revealing how isoform diversity underpins tissue‐specific mitochondrial dynamics and physiological roles of Drp1.

## Graphical abstract

Targeted long‐read sequencing resolves full‐length *DNM1L* (Drp1) isoforms and reveals conserved, tissue‐specific expression patterns across human and mouse tissues. Functional assays show that Drp1 isoforms differ in their ability to drive mitochondrial fission, independent of abundance, with specific exons modulating activity. These findings demonstrate how alternative splicing of *DNM1L* fine‐tunes mitochondrial dynamics in a tissue‐dependent manner.

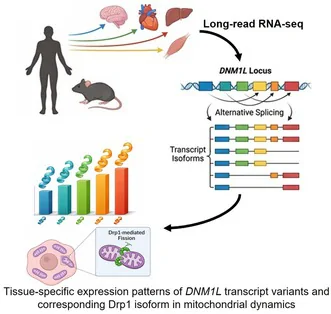

**Abbreviations**
Bcl‐xL: B‐cell lymphoma‐extra large
*DNM1L*
: dyanmin 1‐like gene
Drp1: dynamin‐related protein 1
GTEx: genotype‐tissue expression
GTPase: guanosine triphosphatase
hESC: human embryonic stem cell
IGV: integrated genomics viewer
iPSC: induced pluripotent stem cell
KO: knockout
LV: left ventricle
MEF: mouse embryonic fibroblast
NCBI: National Ccenter for Bbiotechnology Iinformation
SEM: standard error of the mean
TPM: transcripts per million
TSI: tissue specificity index
WT: wild type

## Introduction

Mitochondria are essential organelles within eukaryotic cells, functioning as the primary source of energy production through oxidative phosphorylation. Their morphology is highly dynamic, constantly undergoing processes of fusion and fission [1, 2]. The balance between mitochondrial fusion and fission is crucial for maintaining cellular homeostasis, adapting to various physiological conditions, and responding to cellular stress [3]. Disruption of this balance, particularly through mutations or dysregulation of Dynamin‐related protein 1 (Drp1), a key driver of mitochondrial fission, results in fragmented, dysfunctional mitochondria that can lead to cellular damage or death. Drp1 also plays a pivotal role in essential cellular processes such as proliferation, survival, autophagy and pluripotency, with its absence being embryonically lethal [1, 2, 3, 4]. Despite its importance, the exact mechanisms through which Drp1 influences mitochondrial health remain poorly understood, constituting a significant knowledge gap in mitochondrial biology.

An emerging area of research focuses on the isoforms of Drp1, with eight distinct isoforms identified in humans. These isoforms primarily arise from alternative splicing of exon 3 (encoding the GTPase domain) and exons 16 and 17 (encoding the variable domain) of the *DNM1L* gene [1] (Fig. 1A–C, Table 1). In mice, alternative splicing of exon 1–4 and 16–17 of the mouse *Dnm1l* gene has resulted in the identification of 20 Drp1 isoforms (Fig. 2, Table 2). A study by Strack and colleagues reported tissue‐specific expression of certain Drp1 isoforms in mice. For example, isoforms containing exon 3 (equivalent to human isoform 5, 6 and 8) are highly expressed in brain and skeletal muscle, while those lacking exon 16 but containing exon 17 (equivalent to human isoform 2) are primarily found in the kidney, spleen and heart [5]. Additionally, excessive levels of specific isoforms, such as isoform 5, may be linked to disease pathologies, including brain tumours [6]. Recently, distinct splice variants of Drp1 have been shown to differentially influence cell proliferation, migration, sensitivity to chemotherapeutic agents and tumour growth *in vivo*. The splice variant containing the full‐length variable domain (including exons 16 and 17) plays a more active role in promoting mitochondrial fission. In contrast, the variant lacking exon 16 contributes less significantly to this process [7]. These isoforms display tissue‐specific expression profiles, suggesting that they may play distinct roles in cellular functions [1, 6, 8]. However, the incomplete understanding of their expression patterns, with only a few studies reporting differential expression, coupled with the lack of clarity regarding how each isoform contributes to mitochondrial dynamics, complicates our comprehension of the role of Drp1 in health and disease. Moreover, many studies on Drp1 have failed to clearly specify which isoform was investigated or have overly focused on certain isoforms [1, 8, 9, 10], which may not be representative of the full range of Drp1 activities. This lack of specificity in research has contributed to conflicting findings, hindering the development of effective therapeutic strategies targeting Drp1.

**Fig. 1:**
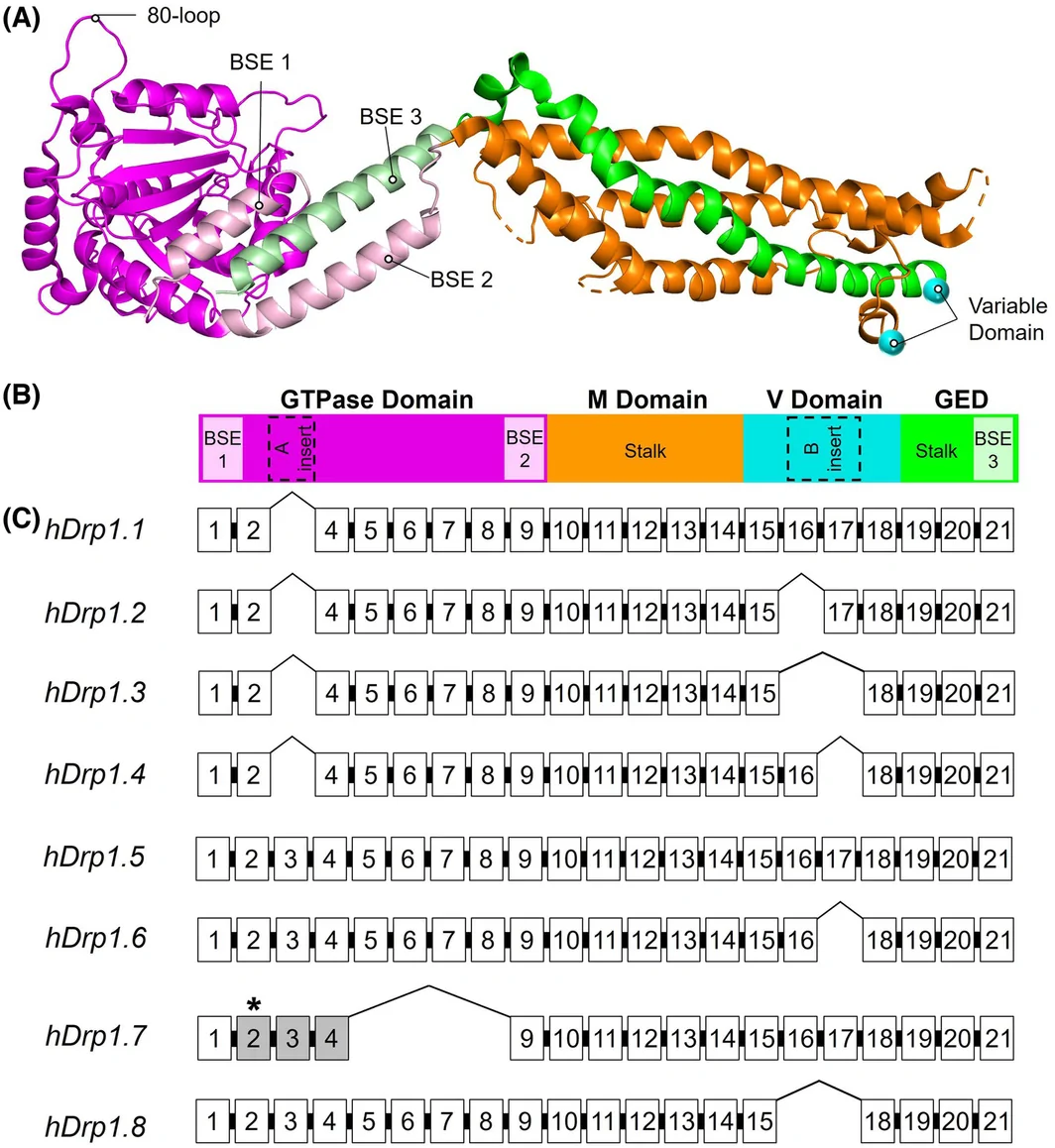
Structure and coding region of human Drp1 isoforms. (A) Cartoon representation of the composite Drp1 model assembled from crystal structures (PDB IDs: 4H1V, residues 1–285; and 4BEJ, residues 282–701). The structure is colour‐coded by domain: the GTPase domain is shown in magenta and includes bundle signalling elements (BSE) 1 and 2 in light pink; the middle (stalk) domain appears in orange; the variable domain is depicted as cyan spheres, representing only the visible N‐ and C‐terminal residues; and the GTPase effector domain (GED) is shown in green, encompassing portions of the stalk and BSE 3 (light green). The figure was generated using the PyMOL Molecular Graphics System v.2.3.2. (B) The linear schematic, colour‐coded to match the structure in panel A, illustrates the domain organisation of Drp1. The A‐ and B‐inserts are depicted as dashed black boxes. (C) Coding regions for human Drp1 isoforms 1–8 are depicted with exon numbering based on the coding sequence of isoform 5 (CCDS61095.1). The GTPase domain is encoded by exons 1–9, the middle (M) domain by exons 10–14, the variable (V) domain by exons 15–18, and the GED by exons 19–21. The A‐insert, located within exon 3, forms part of the GTPase domain, while the B‐insert, spanning exons 16–17, resides within the variable domain. Exons, introns, and domains are not shown to scale. White rectangles represent exons; ˄ indicates skipped exons; * marks an alternative start codon; and grey shading highlights regions encoding alternative amino acid sequences.

**Table 1:** Human DRP1 isoforms and *DNM1L* splice variants.

Isoform ID	Transcript accession	Transcript length (nucleotides)	Transcript protein accession	Transcript protein length (amino acids)
1	NM_012062.5	4514	NP_036192.2	736
2	NM_012063.4	4436	NP_036193.2	710
3	NM_005690.5	4403	NP_005681.2	699
4	NM_001278463.2	4481	NP_001265392.1	725
5	NM_001278464.2	4553	NP_001265393.1	749
6	NM_001278465.2	4520	NP_001265394.1	738
7	NM_001278466.2	4110	NP_001265395.1	533
8	NM_001330380.2	4442	NP_001317309.1	712

**Fig. 2:**
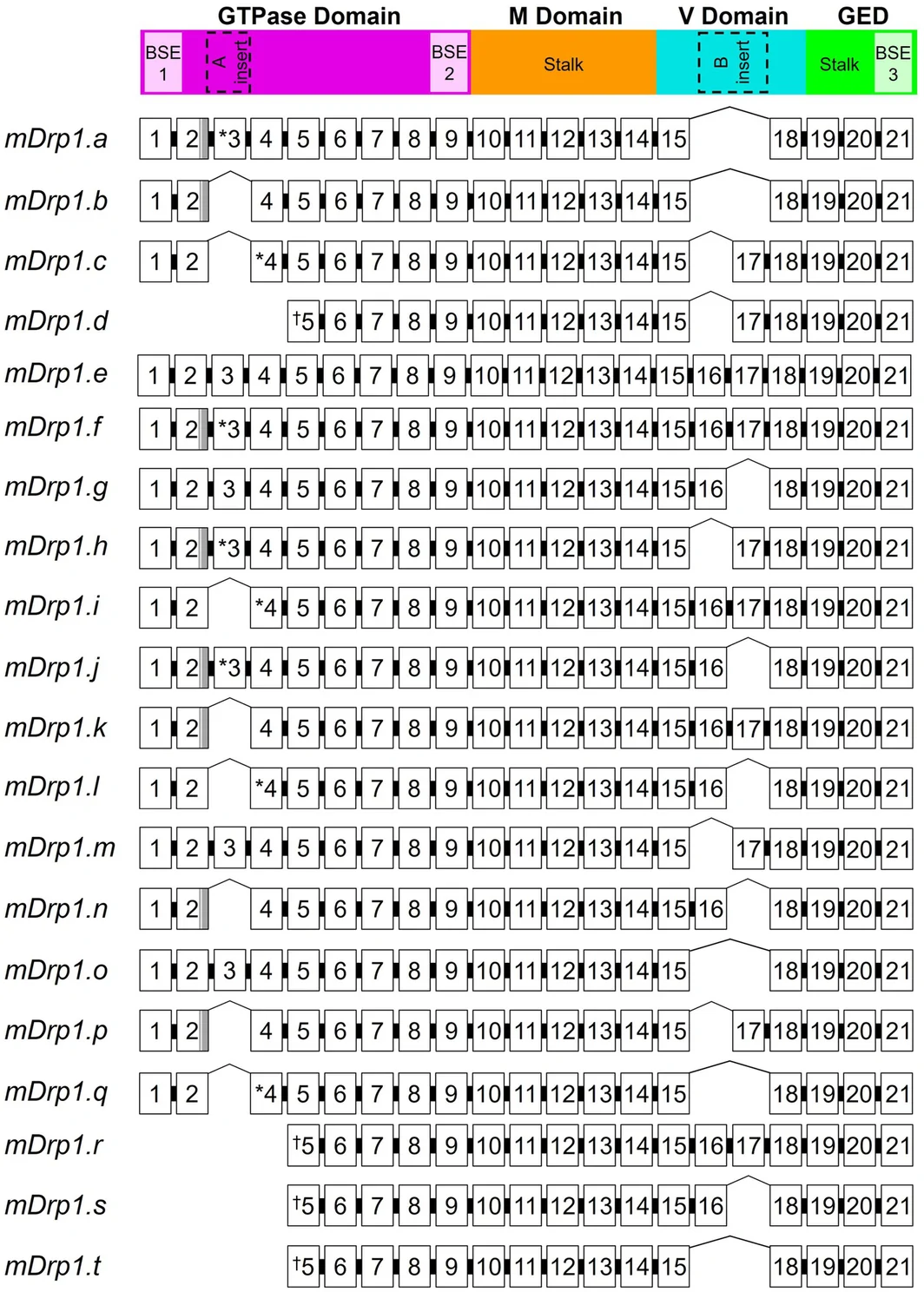
Structure and coding region of mouse Drp1 isoforms. Coding regions of mouse Drp1 isoforms a–t with exon numbering based on the longest isoform, isoform e (mRNA transcript NM_001360007.2, protein NP_001346936.1). The GTPase domain is encoded by exons 1–9, the middle (M) domain by exons 10–14, the variable (V) domain by exons 15–18, and the GTPase effector domain (GED) by exons 19–21. The A‐insert, located within exon 3, forms part of the GTPase domain, while the B‐insert, spanning exons 16–17, resides within the variable domain. Exons, introns, and domains are not shown to scale. White rectangles represent exons. Squares with shaded grey rectangles on exon 2 denote missing amino acid compared to mDrp1.e (GKFQSW) at C‐terminus. * on exon 3 and 4 denote first amino acid is different from isoform e (N > D for exon 3) and (G > R for exon 4). ^†^ on exon 5 denote addition of amino acid (M) at N‐terminus.

**Table 2:** Mouse Drp1 isoforms and *Dnm1l* splice variants.

Isoform ID	Transcript accession	Transcript length (nucleotides)	Transcript protein accession	Transcript protein length (amino acids)
a	NM_152816.4	4023	NP_690029.2	712
b	NM_001025947.3	3984	NP_001021118.1	699
c	NM_001276340.2	4035	NP_001263269.1	716
d^a^	NM_001405252.1	3970	NP_001392181.1	612
d^a^	NM_001276341.2	3790	NP_001263270.1	612
e	NM_001360007.2	4152	NP_001346936.1	755
f	NM_001360008.2	4134	NP_001346937.1	749
g	NM_001360009.2	4119	NP_001346938.1	744
h	NM_001360010.2	4056	NP_001346939.1	723
i	NM_001405253.1	4113	NP_001392182.1	742
j	NM_001405254.1	4101	NP_001392183.1	738
k	NM_001405255.1	4095	NP_001392184.1	736
l	NM_001405256.1	4080	NP_001392185.1	731
m	NM_001405257.1	4074	NP_001392186.1	729
n	NM_001405258.1	4062	NP_001392187.1	725
o	NM_001405259.1	4041	NP_001392188.1	718
p	NM_001405260.1	4017	NP_001392189.1	710
q	NM_001405261.1	4002	NP_001392190.1	705
r	NM_001405262.1	4048	NP_001392191.1	638
s	NM_001405263.1	4015	NP_001392192.1	627
t^b^	NM_001405264.1	3937	NP_001392193.1	601
t^b^	NM_001405265.1	3757	NP_001392194.1	601

^a^ NM_001405252.1 and NM_001276341.2 encode the same isoform d but differ in their 5′ untranslated regions.

^b^ NM_001405264.1 and NM_001405265.1 encode the same isoform t but differ in their 5′ untranslated regions.

The structural differences between Drp1 isoforms, such as variations in protein domains and GTPase activity, may impact their oligomerisation and interactions with other cellular proteins [1, 8, 11, 12, 13]. These differences could affect the accessibility of binding sites for small molecule inhibitors or peptides, influencing their potential as therapeutic agents. Some studies indicate that certain isoforms may be more involved in regulating mitochondrial fission, apoptosis, or other cell functions in specific tissues. For example, isoform 2 has been shown to confer resistance to apoptosis in some models, whereas isoforms containing specific exons, such as isoforms 1 and 6, may interact with anti‐apoptotic proteins like Bcl‐xL [5, 14]. These findings suggest that selectively targeting Drp1 isoforms could open new avenues for disease‐specific treatments, particularly in conditions like neurodegenerative diseases, cardiovascular disorders and cancer.

Given the complexity of Drp1 isoforms generated through alternative splicing and their potential contribution to cellular dysfunction, it is critical to investigate their tissue‐specific expression, structural diversity and functional consequences. Short‐read sequencing has limited full‐length isoform characterisation, obscuring their biological significance. In this study, we applied long‐read transcriptomics together with functional assays to comprehensively define *DNM1L/Dnm1l* isoforms, revealing distinct tissue‐enriched expression patterns and isoform‐dependent differences in mitochondrial fission activity. By establishing a cross‐tissue atlas of *DNM1L/Dnm1l* expression and function in mice, we provide new insights into how Drp1 isoform diversity shapes mitochondrial dynamics, with implications for developing more precise therapeutic strategies for mitochondrial‐related diseases.

## Results

### Capture of full‐length *DNM1L/Dnm1l* transcripts in human and mouse tissues

To investigate transcript diversity of *DNM1L/Dnm1l*, we performed high‐specificity long‐read sequencing using a tailored cDNA synthesis and amplification workflow optimised for full‐length isoform resolution (Fig. 3A). Starting from 300 ng of high‐integrity total RNA (RIN > 8) per sample, reverse transcription was carried out with Maxima H Minus Reverse Transcriptase using a custom oligo(dT) primer and a template‐switch oligo containing a Unique Molecular Identifier (UMI), enabling precise molecule tracking and error correction. A key specificity‐enhancing step involved targeted amplification of *DNM1L/Dnm1l* transcripts using biotinylated primers (HEx21‐R1 for human or MEx21‐R1 for mouse) designed to anneal at exon 21 before the stop codon, a region conserved across all known isoforms. This targeted enrichment strategy, coupled with the use of high‐fidelity KAPA HiFi polymerase and template‐switch‐based amplification, enabled selective capture of full‐length *DNM1L/Dnm1l* transcripts. Biotin‐tagged amplicons were isolated via streptavidin‐coated magnetic beads and re‐amplified using the same exon‐specific primers to ensure robustness and consistency. Purified libraries were sequenced using the PromethION platform from Oxford Nanopore Technologies with native barcoding, and base calling was conducted in super accuracy mode, providing high‐confidence long‐read data. This targeted, UMI‐guided, full‐length approach enabled deep isoform‐resolved analysis of *DNM1L/Dnm1l*, capturing transcript variants with high specificity and minimising amplification bias.

**Fig. 3:**
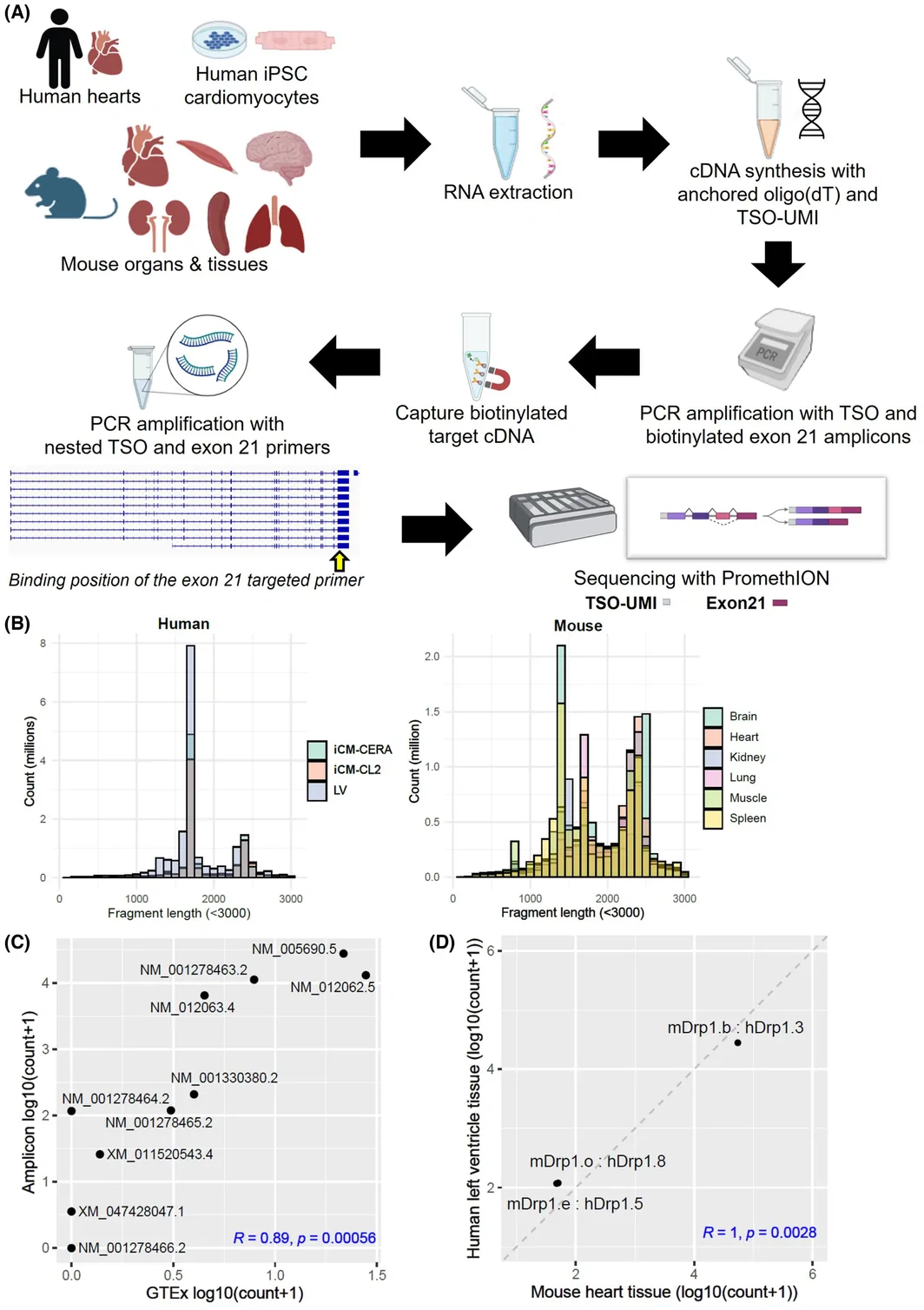
Long‐read transcriptomic profiling and cross‐species comparison of *DNM1L/Dnm1l* isoforms. (A) long‐read transcriptomic sequencing workflow. (B) Read length distributions across biological human and mouse samples. iCM‐CERA (CERA iPSC‐derived cardiomyocytes), iCM‐CL2 (CL2 iPSC‐derived cardiomyocytes), LV (human left ventricle). (C) Scatter plot of expression correlation between amplicon and GTEx datasets (*R* = 0.89, *P* = 0.00056). Transcript expression correlation of human *DNM1L* transcripts between amplicon captured in house data and GTEx data. Expression values were averaged from left ventricle samples only in both datasets, and log10 transformed with offset 1. (D) Correlation of conserved human and mouse Drp1 isoforms selected for experimental validation. The label is shown as (mouse isoform id: human isoform id). mDrp1.b: hDrp1.3 (NM_001025947.3: NM_005690.5), mDrp1.e: hDrp1.5 (NM_001360007.2: NM_001278464.2), mDrp1.o: hDrp1.8 (NM_001405259.1: NM_001330380.2). Expression was calculated by averaging human left ventricle samples and mouse heart tissues, and log 10 transformed with offset 1.

Comprehensive data analysis confirmed the high quality and reproducibility of our targeted long‐read sequencing pipeline. Quality control analyses were performed to confirm the robustness and lack of bias in the amplicon capture and sequencing workflow. The distribution of read lengths across samples and groups showed consistent profiles, with modal lengths corresponding to full‐length *DNM1L/Dnm1l* transcript isoforms, indicating successful amplification and capture of complete intact cDNA (Fig. 3B, Fig. S1A,B). UMI saturation analysis demonstrated near‐complete recovery of unique transcript molecules within each sample, indicating that sequencing depth was sufficient and that PCR duplicates were effectively accounted for by UMI collapsing (Fig. S2A,B). Using human left ventricle samples as a reference, we performed correlation analysis between *DNM1L/Dnm1l* transcript expression levels in our targeted amplicon dataset and public GTEx bulk long‐read RNA‐seq data. The analysis revealed strong concordance (Pearson's *R* = 0.89), supporting isoform proportions are preserved in our targeted sequencing approach (Fig. 3C). Furthermore, several lowly expressed transcripts were only detected using our approach, demonstrating the comprehensive capture of all isoforms of *DNM1L/Dnm1l*. Transcript coverage profiling from the 5′ to 3′ ends confirmed uniform representation across the transcript length with no positional bias, and a clear drop of coverage corresponded to the position of primer location and start of 5′ UTR. This underscores the effectiveness of template‐switching combined with oligo(dT)‐primed reverse transcription (Fig. S3A,B). Similar uniform representation was observed using exonic coverage, except exon 3, 16 and 17, consistent with high‐efficiency capture of full‐length isoforms (Fig. S3C,D). These depleted exons were consistent with the visual inspection of *DNM1L/Dnm1l* in Integrated Genomics Viewer (IGV) in representative samples, which also illustrate clean mapping of reads, absence of spurious alignments, and accurate capture of exon‐exon junctions (Fig. S4). Expression of three selected *DNM1L/Dnm1l* transcript isoforms showed strong correlation between human left ventricle and mouse heart tissues, supporting both technical reproducibility and biological conservation across species (Fig. 3D).

### Transcript variant diversity of *DNM1L* in human cardiomyocytes and heart tissues

Our long‐read RNA sequencing dataset demonstrates robust capability to capture full‐length *DNM1L* transcripts in human iPSC‐derived cardiomyocytes (iCM‐CERA and iCM‐CL2) and human left ventricle, enabling accurate isoform‐level analysis. The on‐target rate for the *DNM1L* gene ranged from 8.8% to 36.7% across samples (Fig. 4A). There were 28 821–133 361 unique UMIs for *DNM1L t*ranscript in each sample (Fig. 4B). Principal component analysis (PCA) based on log_2_‐transformed Transcript unique UMI counts Per Million (log_2_TPM) for *DNM1L* transcripts showed expected sample clustering, with iPSC‐derived cardiomyocytes separating slightly from primary left ventricle samples (Fig. 4C). This suggests that *DNM1L* transcript isoform diversity is associated with cardiac tissue identity and developmental state.

**Fig. 4:**
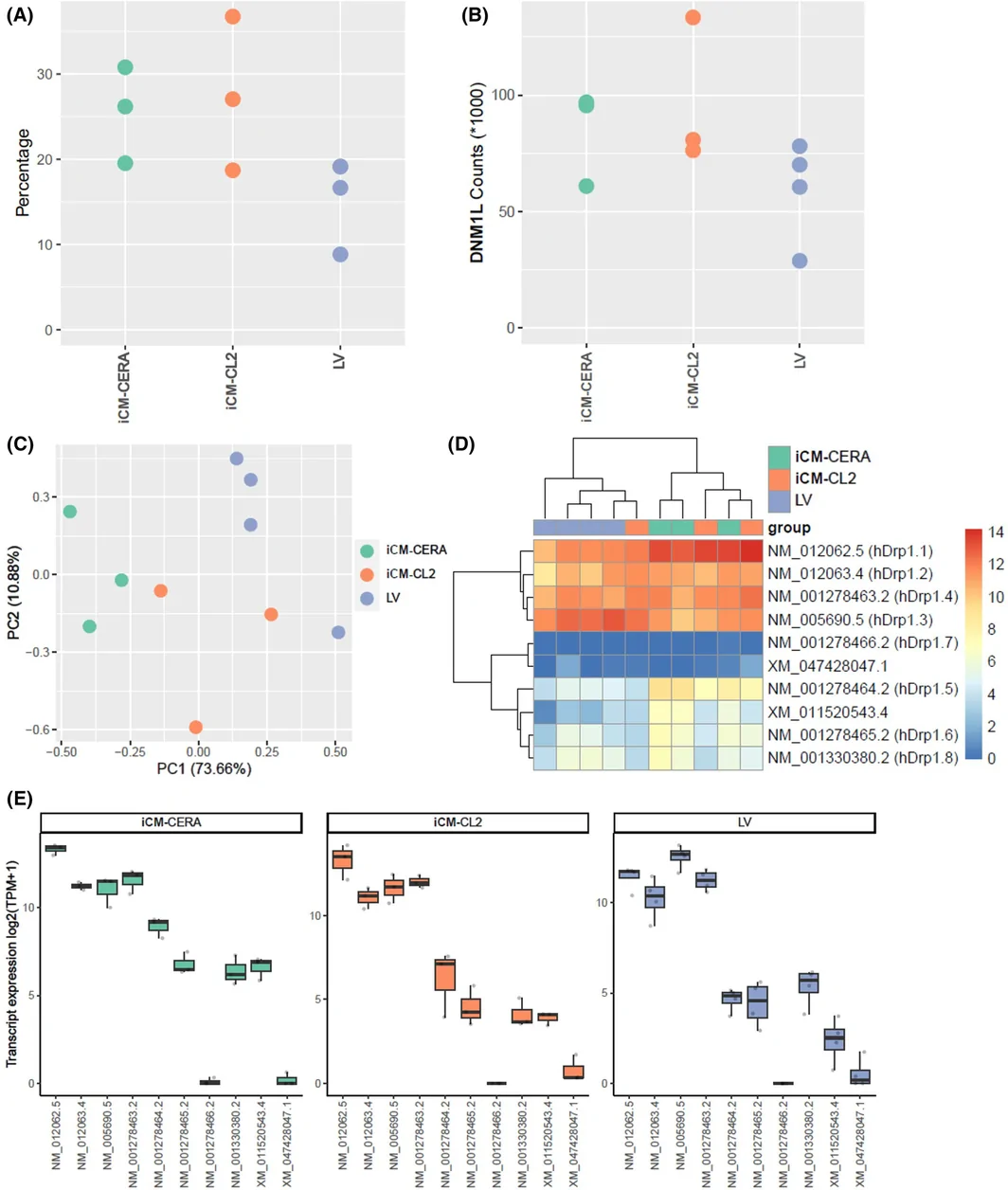
Expression of *DNM1L* transcript variants in human cardiomyocytes and heart tissue. (A) On‐target rate for the *DNM1L* gene. (B) Total unique UMI counts for all *DNM1L* transcripts. (C) PCA plot of iCM‐CERA, iCM‐CL2, and primary left ventricle samples based on *DNM1L* transcripts using log_2_(TPM + 1). (D) Heatmap of *DNM1L* transcript expression [log_2_(TPM + 1)]. (E) Boxplots showing *DNM1L* transcript expression [log_2_(TPM + 1)] in iCM‐CERA, iCM‐CL2, and primary left ventricle samples.

The dataset comprehensively recovered the full set of annotated *DNM1L* transcripts listed in the NCBI record. In both human iPSC‐derived cardiomyocytes and primary left ventricle samples, transcript variant abundance patterns were broadly similar. Isoforms 1, 2, 3 and 4, all lacking exon 3, were the most abundantly expressed across both sample types, followed by moderate expression of isoforms 5, 6 and 8. Isoform 7, missing exon 5 to 8, was consistently detected at low levels. Among the two XM transcripts identified, XM_011520543.4 displayed moderate expression, whereas XM_047428047.1 was expressed at low levels, comparable to isoform 7 (Fig. 4D,E). Together, these results confirm the robust capture of the full spectrum of *DNM1L* transcript variants in both cardiomyocytes and primary heart tissue.

The functional relevance of the observed expression patterns was assessed by measuring the oligomerisation state, GTP‐binding affinity, and GTPase activity of recombinant Drp1 isoforms. Drp1 mediates mitochondrial fission through oligomerisation on the outer mitochondrial membrane. At a concentration of 200 nm, Drp1 isoforms 1, 2, 3, 4, 5, 6 and 8 exhibited comparable oligomerisation behaviour, existing predominately as dimers both in solution (Fig. 5A) and in the presence of the non‐hydrolysable GTP analogue GMPPCP (data not shown). All isoforms bound GDP with low micromolar affinity (Fig. 5B). Among them, isoform 3 showed noticeably weaker GDP binding, as indicated by a higher KD value relative to the other isoforms (Fig. 5B). Analysis of enzymatic activity revealed that isoforms 3, 5 and 8 exhibited the highest enzymatic activity, whereas isoforms 1, 2, 4 and 6 showed comparatively lower activity (Fig. 5C). These findings indicate that transcript abundance does not necessarily correlate with biochemical activity of Drp1 isoforms and highlight the importance of integrating both expression data and biochemical characterisation to understand isoform‐specific contributions to Drp1 function in the cardiomyocytes and heart tissues.

**Fig. 5:**
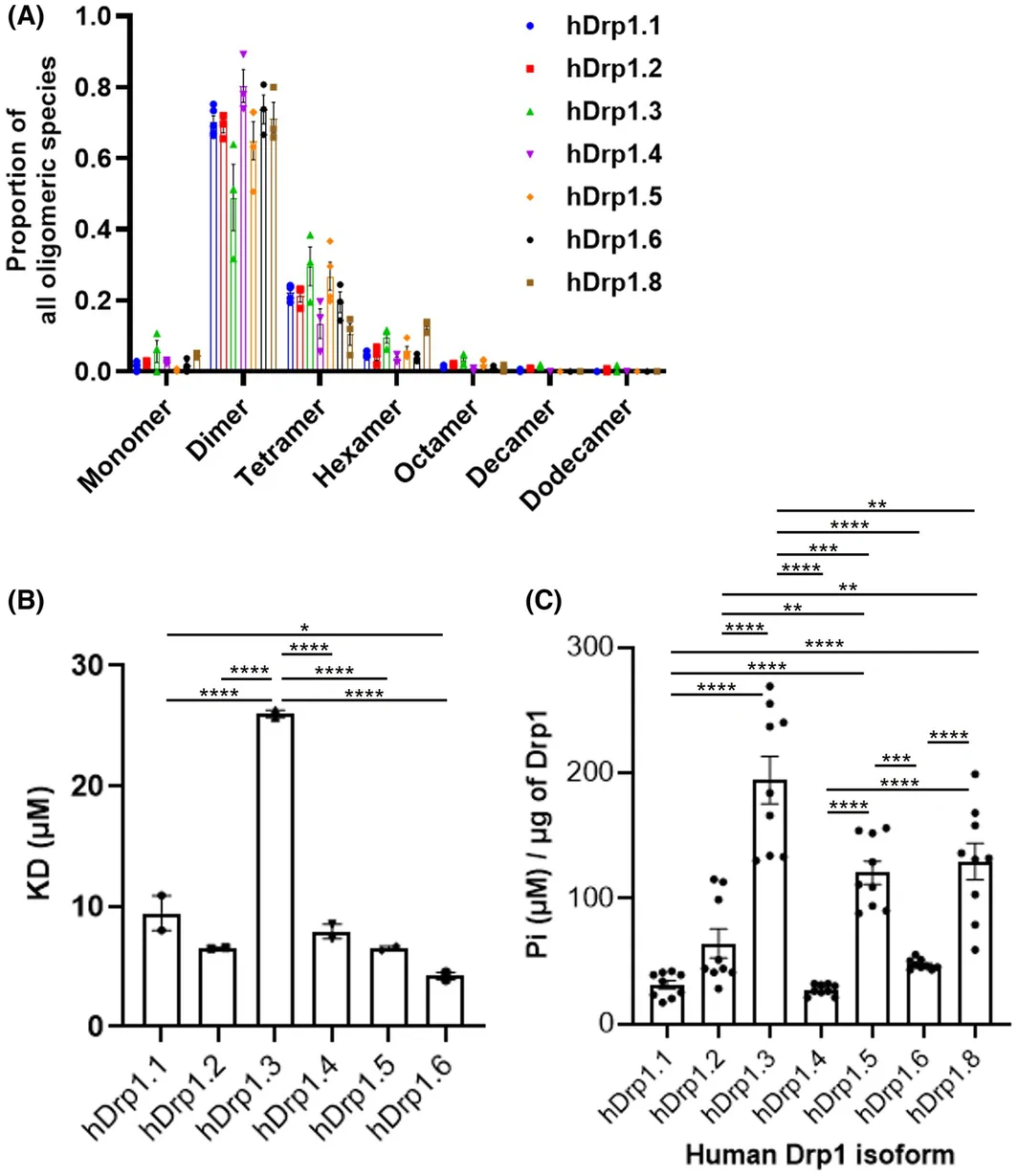
Biochemical analysis of recombinant human Drp1 isoform proteins. Recombinant Drp1 isoforms were expressed, purified and analysed for their oligomerisation state (A), GDP‐binding affinity (B), and GTPase enzymatic activity (C). (A) Oligomerisation analysis showed that all isoforms were predominantly dimeric at a concentration of 200 nm. Measurements were performed in triplicate and independently repeated at least three times. (B) SPR analysis of GDP binding revealed that isoform 3 exhibited weaker binding affinity compared to the other isoforms. Assays were performed in duplicate and independently repeated twice. (C) GTPase activity was quantified by measuring the rate of inorganic phosphate release over time in the presence of GTP. All reactions were conducted under identical buffer and temperature conditions. Assays were performed in triplicate and independently repeated three times. Data are presented as mean ± SEM. *P < 0.05, **P < 0.01, ***P < 0.001, ****P < 0.0001 by one‐way ANOVA with Bonferroni *post hoc* test.

### Long‐read RNA sequencing of *Dnm1l* transcript variants in primary mouse tissues

Long‐read RNA sequencing enabled comprehensive detection of *Dnm1l* isoforms across multiple primary mouse tissues, including brain, heart, kidney, lung, skeletal muscle and spleen. The on‐target rate for the *Dnm1l* gene ranged from 15.8% to 51.8% across samples (Fig. 6A). There were 59 584 to 178 684 unique UMIs for *Dnm1l* transcripts in each sample, providing robust coverage. The dataset recovered the complete set of annotated *Dnm1l* transcripts listed in the NCBI record, encompassing 22 NM (protein‐coding), 14 NR (non‐coding RNA), and one XM (predicted protein‐coding) transcripts, underscoring the sensitivity and completeness of long‐read sequencing in capturing both coding and non‐coding variants across the locus. These results confirm effective detection of all known isoforms, ensuring sufficient depth and diversity for isoform‐resolved analyses.

**Fig. 6:**
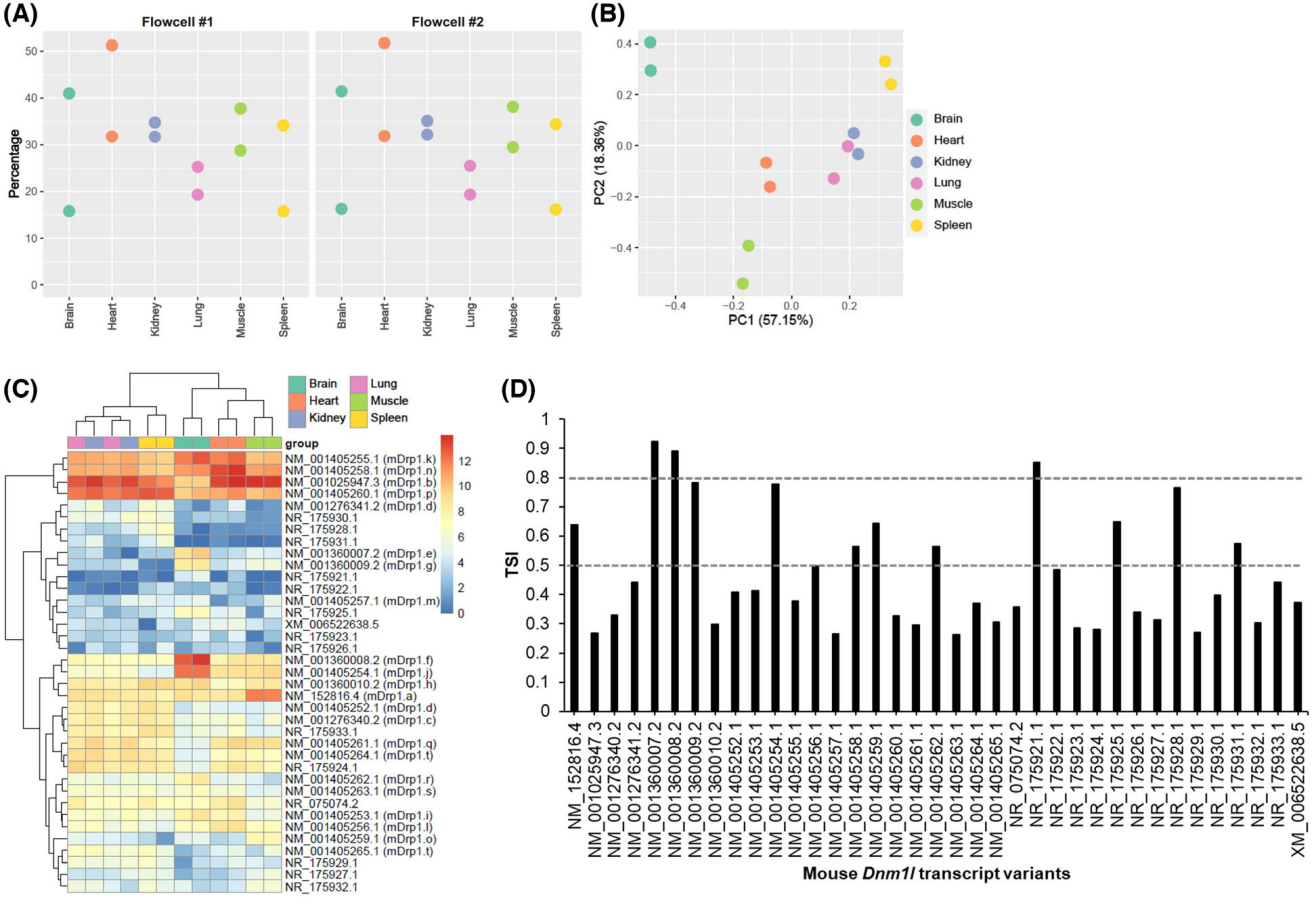
Expression of *Dnm1l* transcript variants in mouse tissues. (A) On‐target rate for the *Dnm1l* gene. (B) PCA plot of mouse brain, heart, kidney, lung, skeletal muscle and spleen samples. (C) Heatmap of *Dnm1l* transcript expression [log_2_(TPM + 1)]. (D) Tissue specificity index (TSI) of all *Dnm1l* transcripts across mouse tissues.

PCA revealed distinct tissue‐specific clustering of *Dnm1l* transcripts (Fig. 6B). Brain and spleen occupied separate regions in the PCA space, while organs rich in epithelial tissues, such as lung and kidney, clustered together. Heart and skeletal muscle were positioned in close proximity, reflecting similarities in their *Dnm1l* transcript profiles. Overall, *Dnm1l* transcript expression contributed substantially to the principal components, suggesting that isoform diversity correlates with tissue identity.

A more detailed analysis of *Dnm1l* transcript isoform expression revealed a spectrum of transcriptional patterns, ranging from broadly expressed to highly tissue‐specific isoforms (Fig. 6C). Hierarchical clustering of tissues and isoforms identified groups of co‐expressed transcripts, consistent with potential functional specialisation of specific *Dnm1l* isoforms. Isoforms e (NM_001360007.2), f (NM_001360008.2), g (NM_001360009.2) and j (NM_001405254.1) were predominantly expressed in brain, suggesting a specialised role in neuronal function. In contrast, isoforms l (NM_001405256.1) and n (NM_001405258.1) were selectively enriched in heart, while isoforms a (NM_152816.4) and o (NM_001405259.1) were relatively enriched in skeletal muscle. Boxplots of individual transcript expression revealed both within‐ and between‐tissue variability, confirming the existence of dominant isoforms with broad expression alongside minor isoforms with restricted, tissue‐specific expression (Fig. S5). Tissue specificity indices (TSI) analysis quantitatively supported these findings, with values near 1 indicating highly tissue‐specific isoforms and values near 0 reflecting broadly expressed transcripts (Fig. 6D).

To explore whether tissue‐specific isoform composition may influence Drp1 activity under pathological conditions, phosphorylation status of Drp1 at Ser616 and Ser637, well‐established surrogate markers of Drp1 activity, in mouse heart and brain tissues isolated from diabetic (*db/db*) mice were evaluated. While this approach does not allow measurement of isoform‐specific activity, it provides an indirect assessment of Drp1 functional status. Our results revealed a significant increase in phosphorylation of Drp1 at Ser616, but not at Ser637, in heart tissues from *db/db* diabetic mice compared with *db/+* controls (Fig. S6). Given that isoforms l and n are selectively enriched in heart tissue, the higher levels of Drp1 Ser616 phosphorylation likely reflects increased activation of these cardiac‐enriched isoforms in the diabetic state. In contrast, no significant differences were observed in phosphorylation at either Ser616 or Ser637 in brain tissues between *db/db* and *db/+* mice (Fig. S6). As brain tissue predominantly expresses isoforms e, f, g and j, these findings suggest that these isoforms may be less responsive to diabetic stress at the level of phosphorylation‐dependent regulation, or that compensatory mechanisms maintain Drp1 activity within a physiological range in the brain.

Together, these analyses demonstrate that long‐read RNA sequencing not only captures the full complement of canonical *Dnm1l* transcripts but also resolves isoform‐specific expression dynamics across tissues. The observed tissue‐enriched expression of select isoforms, together with the tissue‐specific differences in Drp1 phosphorylation in diabetes, suggests that Drp1 isoform diversity may contribute to differential regulation of mitochondrial dynamics across tissues. In particular, increased Drp1 Ser616 phosphorylation in the heart is consistent with increased Drp1 activation and may contribute to altered mitochondrial dynamics in diabetic cardiomyopathy, whereas the absence of phosphorylation changes in the brain points to distinct regulatory control of Drp1 isoforms in neuronal tissues.

### Differential effects of Drp1 isoforms on mitochondrial fission

To investigate the specific contributions of Drp1 isoforms to the regulation of mitochondrial morphology, a Drp1 knockout (KO) mouse embryonic fibroblast (MEF) model was used (Fig. 7). Distinct mouse Drp1 splice variants were individually and stably expressed in these cells to evaluate their ability to rescue mitochondrial fission defects. The isoforms tested included: isoform b, which lacks exons 3, 16 and 17 and contains an incomplete exon 2 and is broadly expressed across tissues; isoform d, which lacks exons 1–4 and 16; isoform e, the full‐length canonical form that is highly expressed in brain; and isoform o, which lacks exons 16 and 17 and is enriched in skeletal muscle. Sequence homology analysis using BLASTp revealed that mouse Drp1 isoforms b, d, e and o exhibited the highest similarity with human Drp1 isoforms 3, 2, 5 and 8, respectively (Table 3). Western blotting revealed similar stable protein expression of mouse isoforms b, e and o in KO MEFs, with expression of isoform d detected at noticeably lower levels (Fig. 7A).

**Fig. 7:**
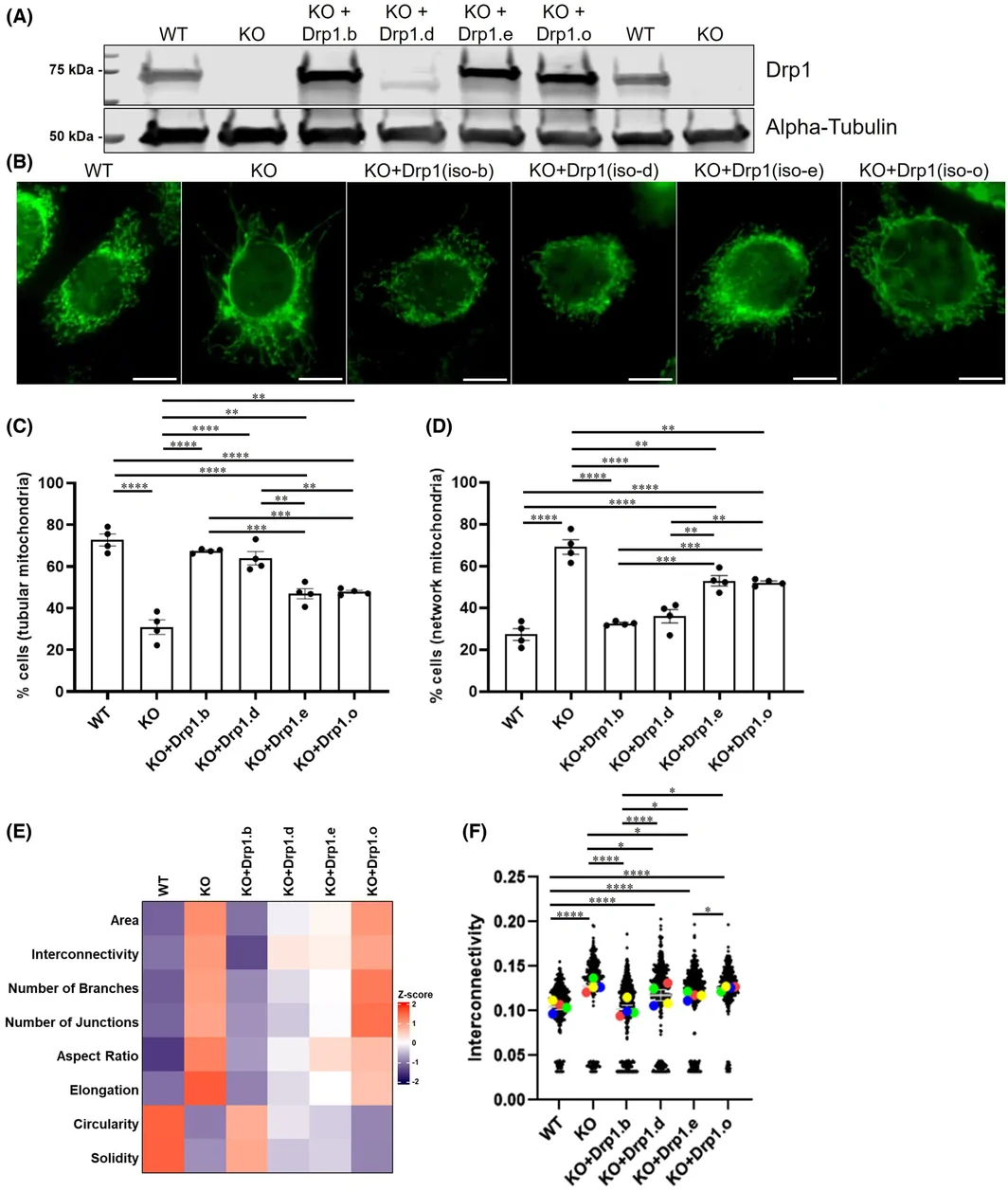
Mitochondrial morphology in MEFs. (A) Protein expression of Drp1 in KO MEFs. (B) Representative fluorescent images of mitochondrial morphology at 600× magnification in MEFs stained with Hsp60 antibody. Scale bar = 10 μm. (C, D) Manual classification quantified the percentage of cells with (C) tubular or (D) network mitochondria in MEFs (wild type, Drp1‐KO, Drp1‐KO re‐expressing Drp1 isoform‐b, isoform‐d, isoform‐e or isoform‐o). (E, F) Automated image analysis was performed in FIJI, with mitochondrial morphology in individual cells quantified following segmentation by adaptive thresholding using the Mitochondrial Analyzer plugin. (E) Heatmap showing whether each condition is above (positive *z*‐score; red) or below (negative *z*‐score; blue) the mean across all conditions (white), with colour intensity representing the number of standard deviations from the mean. (F) Mitochondrial interconnectivity measured in individual cells for each condition. *N* = 4 replicates. Data are expressed as mean ± SEM. *P < 0.05, **P < 0.01, ***P < 0.001, ****P < 0.0001 by one‐way ANOVA with Bonferroni *post hoc* test.

**Table 3:** Comparative sequence similarity of Drp1 isoforms in mouse and human using BLASTp algorithm.

Mouse Drp1 isoforms		Max score (percent identity/per cent query cover)							
Isoform	Transcript protein accession	Human isoform 1	Human isoform 2	Human isoform 3	Human isoform 4	Human isoform 5	Human isoform 6	Human isoform 7	Human isoform 8
a	NP_690029.2	1391 (92.52/100)	1400 (95.57/100)	1408 (97.05/100)	1395 (93.90/100)	1431 (94.26/100)	1434 (95.66/100)	912 (91.84/91)	1446 (98.88/100)
b	NP_001021118.1	1410 (94.16/100)	1410 (97.32/100)	1417 (98.86/100)	1406 (95.59/100)	1391 (92.52/100)	1395 (93.90/100)	912 (91.84/91)	1408 (97.05/100)
c	NP_001263269.1	1418 (94.47/100)	1431 (97.91/100)	1402 (96.51/100)	1398 (94.53/100)	1415 (9347/100)	1394 (93.50/100)	936 (93.47/91)	1399 (95.44/100)
d	NP_001392181.1	1230 (94.82/100)	1243 (98.85/86)	1214 (97.22/85)	1210 (94.89/86)	1230 (94.82/85)	1209 (94.89/84)	935 (93.47/91)	1214 (97.22/84)
e	NP_001346936.1	1478 (96.16/100)	1414 (92.85/100)	1389 (91.52/100)	1449 (94.83/100)	1508 (97.75/100)	1479 (96.42/100)	998 (98.57/91)	1419 (93.11/100)
f	NP_001346937.1	1481 (96.96/100)	1416 (93.59/100)	1390 (92.26/100)	1449 (94.83/100)	1518 (98.66/100)	1488 (97.33/100)	999 (98.57/91)	1430 (93.99/100)
g	NP_001346938.1	1448 (94.83/100)	1394 (93.01/100)	1392 (92.88/100)	1454 (96.24/100)	1479 (96.42/100)	1488 (97.85/100)	970 (96.53/91)	1424 (94.49/100)
h	NP_001346939.1	1416 (93.59/100)	1429 (96.96/100)	1400 (95.57/100)	1394 (93.63/100)	1455 (95.33/100)	1434 (95.39/100)	936 (93.47/91)	1438 (97.37/100)
i	NP_001392182.1	1482 (97.84/100)	1418 (94.47/100)	1394 (93.13/100)	1452 (96.50/100)	1479 (96.80/100)	1450 (95.47/100)	998 (98.57/91)	1390 (92.13/100)
j	NP_001392183.1	1481 (96.93/100)	1396 (93.77/100)	1394 (93.63/100)	1459 (97.02/100)	1488 (97.33/100)	1498 (98.78/100)	970 (96.53/91)	1434 (95.39/100)
k	NP_001392184.1	1488 (98.64/100)	1425 (95.24/100)	1401 (93.89/100)	1459 (97.28/100)	1480 (96.93/100)	1449 (95.59/100)	999 (98.57/91)	1390 (92.26/100)
l	NP_001392185.1	1453 (96.50/100)	1401 (94.66/100)	1398 (94.53/100)	1461 (97.95/100)	1451 (95.47/100)	1456 (96.89/100)	969 (96.53/91)	1394 (93.50/100)
m	NP_001392186.1	1396 (92.85/100)	1427 (96.16/100)	1397 (94.79/100)	1375 (91.36/100)	1445 (94.44/100)	1423 (94.49/100)	936 (93.47/91)	1429 (96.43/100)
n	NP_001392187.1	1459 (97.28/100)	1406 (95.45/100)	1405 (95.31/100)	1469 (98.76/100)	1451 (95.59/100)	1458 (97.02/100)	970 (96.53/91)	1394 (93.63/100)
o	NP_001392188.1	1389 (91.79/100)	1398 (94.79/100)	1405 (96.24/100)	1377 (92.34/100)	1422 (93.38/100)	1425 (94.76/100)	910 (91.84/91)	1437 (97.91/100)
p	NP_001392189.1	1425 (95.24/100)	1437 (98.73/100)	1409 (97.32/100)	1405 (95.31/100)	1416 (93.59/100)	1394 (93.63/100)	936 (93.47/91)	1400 (95.57/100)
q	NP_001392190.1	1395 (93.40/100)	1402 (96.51/100)	1410 (98.01/100)	1398 (94.80/100)	1391 (92.40/100)	1394 (93.78/100)	911 (91.84/91)	1406 (96.91/100)
r	NP_001392191.1	1294 (98.74/86)	1230 (94.82/86)	1206 (93.25/85)	1264 (97.17/86)	1293 (98.74/85)	1264 (97.17/84)	998 (98.57/91)	1205 (93.25/84)
s	NP_001392192.1	1265 (97.17/86)	1211 (95.05/86)	1210 (94.89/85)	1273 (98.88/86)	1264 (97.17/85)	1273 (98.88/84)	970 (96.53/91)	1209 (94.89/84)
t	NP_001392193.1	1207 (93.56/86)	1215 (97.22/86)	1223 (99.00/85)	1210 (95.21/86)	1205 (93.56/85)	1209 (95.21/84)	911 (91.84/91)	1222 (99.00/84)

*Note:* The colour shades indicate the top three "Max score" for each isoform.

Mitochondrial morphology was assessed using both manual visual classification and automated AI‐based analysis. Manual classification quantified the percentage of cells displaying predominantly tubular or networked mitochondria as a simplified binary readout of fission activity. Using this approach, Drp1‐KO MEFs exhibited significantly lower percentage of cells with tubular mitochondria, reflecting reduced mitochondrial fragmentation, compared to Drp1‐wild‐type (WT) MEFs. Re‐expression of isoform b or isoform d fully restored mitochondrial fragmentation, indicating robust functional capacity despite the relatively low apparent expression of isoform d. In contrast, re‐expression of isoform e and isoform o only partially rescued the fission defect (Fig. 7B,D).

Automated analysis showed partial concordance with manual classification across experimental groups. Groups exhibiting pronounced morphological remodelling, such as Drp1‐WT versus Drp1‐KO MEFs, demonstrated agreement between methods. However, in groups with subtler phenotypic differences, AI‐based analyses revealed method‐dependent outcomes that varied according to the specific morphological parameters assessed. For example, analysis of mitochondrial interconnectivity indicated that re‐expression of isoform b fully restored mitochondrial fragmentation, whereas isoforms d and e produced a partial restoration that was less robust than isoform b. In contrast, re‐expression of isoform o failed to restore mitochondrial fragmentation in Drp1‐KO MEFs (Fig. 7E,F). Comprehensive mitochondrial morphology profiling incorporating multiple parameters related to mitochondrial size, shape and network connectivity revealed similar trends across experimental groups (Fig. 7E, Fig. S7). Together, these results demonstrate that distinct Drp1 isoforms exert differential effects on mitochondrial fission. Notably, isoform b, which lacks both the A‐insert within the GTPase domain (encoded by exon 3) and the B‐insert within the variable domain (encoded by exons 16 and 17), was more effective in restoring fission than the full‐length isoform e or the B‐insert‐truncated isoform o, both of which retain an intact GTPase domain. Collectively, these findings suggest that inclusion of exons 2 and/or 3 may exert an inhibitory influence on Drp1‐mediated mitochondrial fission.

## Discussion

Drp1, encoded by the *DNM1L* gene, is an evolutionarily conserved member of the dynamin family and a key regulator of mitochondrial fission. It is ubiquitously expressed, with particularly high levels in mitochondria‐rich tissues such as the brain and heart. Dysregulation of Drp1 has been implicated in various pathophysiological conditions, including neurodegenerative diseases, cardiovascular disorders, and cancer [1]. Despite the established importance of Drp1 in mitochondrial dynamics and cell fate decisions, the diversity and functional significance of Drp1 isoforms remain incompletely understood. This knowledge gap is largely due to the limitations of conventional transcriptomic approaches. Mainstream short‐read RNA sequencing, which involves RNA fragmentation prior to sequencing, is poorly suited to capturing full‐length transcripts and accurately resolving transcript structures, particularly for variants involving widely spaced exons, such as the connections between exon 2–4 and exon 16–17 in *DNM1L*. Even advanced computational tools struggle to reconstruct complete isoform architectures from fragmented reads [15, 16], making it difficult to accurately determine the expression levels and specific functions of individual Drp1 isoforms under various pathophysiological conditions.

To overcome these limitations, the present study employs long‐read sequencing technologies that preserve full‐length RNA molecules, enabling high‐resolution transcript‐level analysis. Using targeted Nanopore native barcoded sequencing, we generated a comprehensive transcriptomic profile of *DNM1L* transcript variants in both human and mouse tissues. This approach allows us to accurately identify and quantify full‐length *DNM1L* transcript variants, offering new insights into their potential roles in health and disease. Our analysis focused on alternative splicing events in exons 1–4, 16, and 17, which give rise to several coding variants annotated in the NCBI record [5, 6, 7, 14, 17, 18, 19]. Previous PCR‐based studies reported tissue‐specific expression of *DNM1L* transcript variants with alternative splicing of exons 16 and 17 in both healthy tissues and human brain tumours [6, 7]. By contrast, our long‐read dataset captured isoforms spanning all 21 exons, revealing tissue‐enriched rather than strictly tissue‐specific expression of *DNM1L* transcript variants. For example, in mouse brain tissue, isoforms f, j, k and n were expressed at higher levels than other isoforms. When expression was compared across tissue types, isoforms e, f, g and j, each containing exon 3, were preferentially enriched in brain, suggesting a specialised neuronal role for exon 3‐containing *Dnm1l* transcript variants. This expression pattern aligns with previous findings by Strack and colleagues, who reported predominant brain expression of exon 3‐containing variants [5], and by Uo *et al*., who identified neuron‐specific *Dnm1l* transcript variants incorporating exon 3 [14]. Interestingly, while Uo *et al*. suggested that exon 17‐containing variants are absent in neurons [14], we observed substantial expression of isoforms e, f, k and p, which contain exon 17, in mouse brain tissue. This discrepancy may reflect differences in sample composition where our dataset represents brain tissue containing diverse cell populations, whereas Uo and colleagues examined isolated neuronal cells.

In the heart, *Dnm1l* transcript variants l and n, which contain exon 16 but lack exons 3 and 17, are expressed at relatively higher levels compared with other tissue types in our dataset. This pattern contrasts with a previous report that described predominant expression of isoforms lacking exon 16 but containing exon 17 in the heart, kidney and spleen [5]. Consistent with that study, however, we also observed that isoforms such as c and p, which lack exon 16 but include exon 17, were more abundant in the kidney and spleen. Notably, mouse *Dnm1l* transcript variants b (homologous to human isoform 3), k (homologous to isoforms 1 and 4), p (homologous to isoform 2) and n (homologous to isoform 4) were highly expressed in mouse heart tissue. This mirrors the strong expression of corresponding isoforms 1–4 in human heart tissue and iPSC‐derived cardiomyocytes, suggesting that the regulation of these isoforms is conserved across species. Our findings reinforce the broader principle that *Dnm1l* transcript isoform diversity is functionally meaningful. While expression patterns show that isoforms are not restricted to a single tissue, the relative abundance and exon composition point to tissue‐preferential functions. Such distribution suggests that isoform‐specific structural differences adapt mitochondrial dynamics to the metabolic and structural requirements of different tissues.

Emerging evidence indicates that Drp1 isoforms exert distinct roles in apoptosis and mitochondrial morphology. Strack *et al*. demonstrated that MEFs lacking endogenous Drp1, as well as MEFs expressing Drp1‐001 (lacking exons 3 and 16), were significantly less sensitive to staurosporine compared with MEFs expressing Drp1‐000 (lacking exons 3, 16, and 17) or Drp1‐011 (lacking exon 3). This reduced sensitivity was associated with a more fused mitochondrial network and a lower rate of apoptosis, suggesting that Drp1‐001 (equivalent to hDrp1.2 and mDrp1.d) may promote cell survival by modulating mitochondrial dynamics [5]. Similarly, Uo *et al*. [14] showed that only isoforms containing both exons 16 and 17 could interact with the anti‐apoptotic protein Bcl‐xL, underscoring isoform‐specific protein–protein interactions in apoptotic regulation.

Our findings further support a model in which alternative splicing of exon 3 acts as a regulatory constraint on Drp1‐mediated mitochondrial fission. Isoforms lacking exon 3 (b and d) consistently exhibited greater fission competence, whereas exon 3‐containing isoforms (e and o) displayed attenuated or loss of activity, suggesting that inclusion of the exon 3‐encoded A‐insert modulates the efficiency of Drp1‐driven membrane remodelling. This interpretation is consistent with prior evidence implicating exon 3 in regulation of GTPase activity and protein interactions [8, 14] supporting the idea that exon 3‐containing isoforms fine‐tune, rather than a simple on/off mechanism of Drp1 function. Minor discrepancies between categorical and continuous morphology analyses likely reflect biological nuance, as graded changes in mitochondrial connectivity may not be fully captured by binary classification. The preferential enrichment of isoform b in brain tissue, which also lacks the B‐insert within the variable domain, further supports the idea that combinatorial splicing imposes structural or regulatory constraints that fine‐tune Drp1 activity across a dynamic range suited to the high metabolic demands and complex mitochondrial dynamics required for neuronal activity and plasticity. Although overexpression studies indicate that exon 3 does not determine Bcl‐xL binding, SUMOylation or intracellular localisation [14], its contribution to GTPase modulation highlights the need for further functional characterisation under physiological conditions.

In cancer, Drp1 isoform regulation is also critical. Javed *et al*. reported that high expression of a splice variant lacking exon 16 (Drp1(−/17)) is associated with poor prognosis in ovarian cancer patients [7]. Unlike the canonical exon 16/17‐containing isoform, which localises to mitochondrial fission puncta and promotes apoptosis, Drp1(−/17) mislocalises to microtubules [5, 7], leading to mitochondrial hyperfusion, enhanced oxidative metabolism, and resistance to pro‐fission stimuli. These changes support tumour growth, survival, and chemoresistance, and manipulation of endogenous isoform ratios confirmed that Drp1(−/17) promotes proliferation and migration, whereas Drp1(16/17) limits tumour progression [7]. Interestingly, our *in vitro* assays did not fully align with these observations. Specifically, isoforms lacking exon 16 (hDrp1.3 and hDrp1.8) displayed significantly higher GTPase activity compared with exon 16‐containing isoforms (hDrp1.1, hDrp1.4 and hDrp1.6). While these results do not contradict previous reports that exon 16‐deficient isoforms mislocalise to microtubules and impair mitochondrial fission, they indicate that such isoforms possess elevated GTPase activity, which would theoretically be expected to facilitate, rather than suppress, mitochondrial fission. Thus, while clinical evidence points to a pro‐tumorigenic role of exon 16‐lacking variants, their precise mechanistic contribution to Drp1 enzymatic activity and mitochondrial dynamics remains to be clarified. Taken together, the relative abundance of exon 3‐, exon 16‐ and exon 16/17‐containing variants emerges as a key determinant of mitochondrial morphology, apoptosis and disease outcomes.

Our study demonstrates that Drp1 (*Dnm1l*) isoforms exhibit distinct GTPase activities and mitochondrial fission capacities, with expression patterns ranging from broadly expressed to highly tissue specific. By leveraging long‐read RNA sequencing, we resolved full‐length transcripts and captured a comprehensive isoform landscape, revealing both shared and tissue‐restricted expression programs that reflect the specialised regulation of mitochondrial dynamics across cell types. These findings underscore the functional complexity of Drp1, showing that alternative splicing shapes isoform‐specific roles in mitochondrial fission, apoptosis, metabolism and disease. Rather than acting solely as a general mediator of mitochondrial division, Drp1 functions in a nuanced, isoform‐dependent manner, with distinct contributions to cellular homeostasis and pathology. While the present study focused on annotated *Dnm1l* transcripts listed in the NCBI reference record, future investigations should leverage long‐read sequencing technologies to uncover novel isoforms that are not yet captured in current annotations. Characterising these variants will provide a more comprehensive understanding of isoform‐specific regulation, structural features, and interaction networks, which will be critical for elucidating their physiological roles and for evaluating the therapeutic potential of selectively targeting individual isoforms in neurodegeneration, cardiovascular disease and cancer.

## Materials and methods

### Animal tissues

Animal tissue sample collection and experimental procedures were approved by the Animal Ethics Committee of St. Vincent's Hospital (AEC 027/22; Victoria, Australia) and were conducted in accordance with the Australian National Health and Medical Research Council guidelines for the care and maintenance of animals. All experiments were performed under the approved animal licence number (AEC 027/22; Victoria, Australia).

Adult male C57BL/6, B6.BKS(D)Lepr db/J heterozygous (db/+) and homozygous (db/db) mice (8 weeks old) were bred in‐house at the BioResources Centre, St Vincent's Hospital (Melbourne, Australia). Animals were maintained in a pathogen‐free facility under a 12‐h light/dark cycle. Mice were group‐housed where space permitted to promote normal social behaviour and reduce stress, while animals exhibiting aggression were singly housed to prevent injury. Environmental enrichment, including plastic tubes for hiding and exploration, was provided in all cages. Mice were euthanised via cervical dislocation. Tissues and organs were promptly harvested and snap‐frozen in liquid nitrogen. Total RNA was extracted using the Aurum™ Total RNA Fatty and Fibrous Tissue Kit (BioRad, Hercules, CA, USA). Briefly, tissues were immersed in 1 mL of RNAlater™ Stabilization Solution (Thermo Fisher Scientific, Waltham, MA, USA) and placed on ice. They were then cut into 3 mm^3^ pieces and mechanically homogenised using a TissueRuptor (Qiagen, Hilden, Germany) for 30–60 s in PureZOL lysis reagent. The homogenate was incubated at room temperature for 5 min and subsequently centrifuged at 12 000 ***g*** for 10 min at 4 °C to pellet insoluble debris. After phase separation, the aqueous phase was processed through a silica spin column. On‐column DNase I digestion was performed to remove genomic DNA. Total RNA was eluted in 40 μL of RNase‐free water and stored at −80 °C.

### Human heart tissues

Donor hearts were obtained from the Sydney Heart Bank, which is approved by the Ethics Committee of The University of Sydney (USYD #2021/122). All procedures conformed to the principles outlined in the Declaration of Helsinki and were conducted with the understanding and written consent of each subject. The hearts were deemed suitable for heart transplantation but unable to be transplanted for reasons including transportation logistics, immune incompatibility, and donor‐recipient mismatch in size. Samples were collected between 1990 and 2010 and were procured as previously described [20, 21, 22]. The hearts underwent formal pathological examination and were deemed structurally normal. Left ventricle (LV) samples were snap‐frozen in liquid nitrogen (−196 °C) within 15 min of harvest. Approximately 40 mg of frozen heart tissues (LV samples from male donors aged 21–56‐year‐old) were transferred to a 2 mL tube containing one 5 mm stainless steel bead (Qiagen) and was disrupted and homogenised in TRIzol reagent (Invitrogen, Carlsbad, CA, USA) using the TissueLyser LT (Qiagen). RNA extraction was then performed according to TRizol manufacturer's instructions. The extracted RNA was then DNase treated using the Qiagen RNAse‐Free DNase Set and subsequently purified using the RNase Mini kit (Qiagen) according to manufacturer's instructions. The concentration and quality of the RNA were assessed using a Nanodrop and the integrity of the RNA was assessed using an RNA Nano Chip on an Agilent Bioanalyzer.

### Cell culture

Human induced pluripotent stem cell (iPSC) lines human iPS‐Foreskin‐2 [23] and CERA007c6 (CERA) [24] were used. The iPS‐Foreskin‐2 (CL2) line (RRID:CVCL_C431) is a human iPSC line derived from foreskin fibroblasts. The CERA007c6 line has been previously was generated and previously described; its identity and pluripotency were validated by expression of OCT4 and TRA1‐60 and by differentiation into derivatives of all three germ layers, and its mitochondrial genotype has also been reported [24, 25]. Human iPSCs were maintained on vitronectin‐coated plates in TeSR‐E8 medium (STEMCELL Technologies, Vancouver, BC, Canada) according to the manufacturer's protocol. All cultures were routinely tested for mycoplasma contamination using PCR‐based assays and were confirmed to be mycoplasma‐free. Human cardiomyocytes were derived from iPSCs using a previously described method [26, 27]. Briefly, iPSCs were seeded onto hESC‐qualified Matrigel (Corning, NY, USA) coated plates at a density of 1.25 × 10^5^ cells·cm^−2^ in TeSR‐E8 medium supplemented with 10 μm Y‐27632 (Abcam, Cambridge, UK). After 48 h, medium was replaced with RPMI 1640 basal medium (Thermo Fisher Scientific) containing B‐27 without insulin supplement (Thermo Fisher Scientific), growth factor reduced Matrigel (1 : 60 dilution; Corning), and 10 μm CHIR99021 (STEMCELL Technologies) (designated as day 0). After 24 h, medium was replaced with RPMI 1640 basal medium containing B‐27 without insulin supplement for 24 h. On day 2, medium was replaced with RPMI 1640 basal medium containing B‐27 without insulin supplement and 5 μm IWP2 (Tocris Bioscience, Bristol, UK) for 72 h. From day 5 onwards, cells were cultured in RPMI 1640 basal medium containing B‐27 supplement (Thermo Fisher Scientific) and 20 ng·mL^−1^ L‐ascorbic acid 2‐phosphate sesquimagnesium salt hydrate (Sigma‐Aldrich, Burlington, MA, USA) (referred to as CM medium), and the medium was refreshed every 2–3 days. On day 12, cardiomyocytes were dissociated into single cells and split at a 1 : 4 ratio onto hESC‐qualified Matrigel coated plates in DMEM/F‐12 GlutaMAX media (Thermo Fisher Scientific) supplemented with 20% foetal bovine serum (Sigma‐Aldrich), 100 μm 2‐mercaptoethanol (Thermo Fisher Scientific), 100 μm non‐essential amino acids (Thermo Fisher Scientific), 50 U·mL^−1^ penicillin/streptomycin (Thermo Fisher Scientific), and 10 μm Y‐27632. At day 13, medium was replaced with CM medium. From days 14 to 19, cardiomyocytes were enriched to > 90% cardiac troponin T‐positive cells by culture in glucose‐free DMEM medium (Thermo Fisher Scientific) containing 4 mm sodium L‐lactate (Sigma‐Aldrich). Total RNA was isolated from enriched day 19–23 iPSC‐derived cardiomyocytes using the Illustra RNAspin Mini Kit (Cytiva, Marlborough, MA, USA) according to the manufacturer's instructions. DNase I enzyme was used to remove genomic DNA. RNA was eluted (twice) off the column in 40 μL of water, quantified using a 2000c Thermo Nanodrop (Thermo Fisher Scientific) spectrophotometer and stored at −80 °C. RNA quality and integrity were assessed using the Agilent TapeStation system, and only samples with acceptable RNA integrity values were included for downstream analysis.

### cDNA synthesis and nanopore sequencing

The process of cDNA synthesis and long‐read sequencing using Nanopore platform is summarised in Fig. 3A. For each sample, 300 ng of high‐quality total RNA (RIN > 8) was reverse transcribed using Maxima H Minus Reverse Transcriptase (Thermo Fisher Scientific) in a 22.5 μL reaction. The reaction included an oligo(dT) primer NaVNP:

5′‐ACTTGCCTGTCGCTCTATCTTCTTTTTTTTTTTTTTTTTTTTVN‐3′,

a template‐switch oligo with unique molecular identifier 10XTSOUMI:

5′‐AAGCAGTGGTATCAACGCAGAGTACANNNYRNNNYRNNNYRNNNVTrGrGrG‐3′

and RNaseOUT (ThermoFisher Scientific). Reverse transcription was performed at 42 °C for 90 min, followed by enzyme deactivation at 85 °C for 5 min. The resulting cDNA was purified using 0.7x SPRIselect beads (Beckman Coulter, Brea, CA, USA) and eluted in 16 μL. The cleaned cDNA was amplified using KAPA HiFi HotStart ReadyMix (Roche, Basel, BS, Switzerland). Initial denaturation was performed at 95 °C for 3 min, followed by 17 cycles of 98 °C for 20 s, 62 °C for 15 s, 72 °C for 3 min and a final extension at 72 °C for 5 min. The reaction included biotinylated HEx21‐R1: 5′‐/5Biosg/AAGATGAGTCTCCCGGATTTCAGC‐3′ or MEx21‐R1: 5′‐/5Biosg/AAGATGAGTCTCTCGGATTTCAGCA‐3′ primers targeting exon 21 in human and mouse *DNM1L/Dnm1l* transcripts, respectively, and ScDNARP: 5′‐AAGCAGTGGTATCAACGCA‐3′ for TSO binding. Amplified products were cleaned using 0.45x SPRIselect beads and eluted in 12 μL. Biotinylated amplicons were captured using 5 μL of Dynabeads M270 Streptavidin (Thermo Fisher Scientific) according to manufacturer instructions. The beads were resuspended in 12 μL. The captured cDNA was re‐amplified with KAPA HiFi HotStart ReadyMix using.

Ex21‐R1 (human): 5′‐AAGATGAGTCTCCCGGATTTCAGC‐3′ or
MEx21‐R1‐2 (mouse): 5′‐AAGATGAGTCTCTCGGATTTCAGCA‐3′ and
cDNARP: 5′‐AAGCAGTGGTATCAACGCAGAG‐3′ primers.

Thermal cycling conditions were the same as above. Amplified products were cleaned with 0.45x SPRIselect and eluted in 20 μL. Libraries were prepared using the Native Barcoding kit (SQK‐NBD114.24, Oxford Nanopore Technologies, Oxford, UK) and loaded onto a PromethION FLO‐PRO114M flow cell for sequencing. Base calling was performed with MinKNOW 23.11.7 super accuracy mode. All oligos were purchased from Integrated DNA Technologies (Coralville, IA, USA).

### Bioinformatic analysis of nanopore‐targeted sequencing

Basecalled Nanopore FASTQ files were processed with flexiplex (v1.01) [28] to remove barcodes and identify UMI sequences, then mapped to the genome with minimap2 (v2.26‐r1175) [29]. For mouse, the GRCm39 genome and NCBI RefSeq annotation (GCF_000001635.27) were used; for human, the GRCh38 genome and NCBI RefSeq annotation (GCA_000001405.15) were used. On‐target rate was calculated using the reads mapping to *DNM1L/Dnm1l* gene region divided by the total number of mappable reads. UMI‐tools (v1.1.6) [30] was used to remove PCR duplicates based on UMIs and genomic coordinates for reads mapped to the *DNM1L/Dnm1l*‐containing chromosome. Saturation analysis was performed by down sampling reads mapped to the *DNM1L/Dnm1l* ‐containing chromosome and performing UMI‐tools deduplication on a subset of the data. Transcript quantification was performed using Bambu (v3.6.0) [31] with UMI‐tools deduplicated BAM files and the corresponding annotation. Seven left‐ventricle samples were downloaded from GTEx [32], aligned to the human genome and quantified directly with Bambu.

To generate transcript coverage plots, we extracted reads mapped to the Drp1‐containing chromosome and realigned them to the *DNM1L/Dnm1l* cDNA sequences. The FLAMES::get_coverage function (v2.1.9) [33] was run on the realigned BAMs to generate transcript coverage plots. To generate genomic coverage plots, we used deepTools (v3.5.5) [34] on genomic BAM files and summarised the regions using a GTF file containing all exons. BAM files were visualised in IGV to produce representative track views.

The tissue specificity index (TSI) [35] for each transcript was calculated as previously described. Briefly, TSI was calculated using the following formula:

$$\mathrm{TSI}=\frac{\max_{1\leq i\leq n}\left(x_{i}\right)}{\sum_{i=1}^{n}x_{i}}$$

*xi* was the average expression (TPM) in a given tissue, and *n* was the number of tissues. Transcripts were then categorised as tissue‐specific (TSI ≥ 0.8), broadly expressed (TSI < 0.5), or biased toward a group of tissues (0.5 ≤ TSI < 0.8). The scripts for analysis are available on GitHub: https://github.com/alexyfyf/Drp1_targeted_ONT.

### Generation of stable Drp1 isoform expression in mouse embryonic fibroblasts (MEFs)

cDNAs encoding for mouse *Dnm1l* isoform b (NM_001025947.3), isoform d (NM_001405252.1), isoform e (NM_001360007.2) and isoform o (NM_001405259.1) were custom synthesised by Gene Universal with C‐terminal FLAG‐tags and cloned into pLeGO‐iG2 using EcoRI/NotI restriction sites. All constructs were sequence‐verified. Drp1 wildtype and knockout (KO) MEFs were kindly provided by Professor Hiromi Sesaki (Johns Hopkins University, USA) [36, 37]. Stable expression of Drp1 isoforms, each bearing a COOH‐terminal FLAG tag, was achieved in Drp1 KO MEFs via lentiviral transduction using the LeGO‐iG2 vector system. Lentiviral particles were produced by co‐transfecting HEK293T cells with the envelope plasmid pCMV‐VSV‐G (Addgene #8454) and the packaging plasmid psPAX2 (Addgene #12260), following a previously described protocol [38]. Drp1 KO MEFs were transduced with 0.45 μm‐filtered viral supernatant in the presence of polybrene, and successfully transduced cells were enriched based on EGFP expression using fluorescence‐activated cell sorting.

### Western blot analysis

Drp1 expression was analysed by western blotting. Proteins from MEFs was extracted with 1% (v/v) Triton X‐100 lysis buffer (50 mm Tris, pH 7.6, 150 mm NaCl, 10% glycerol) supplemented with 1 mm EDTA, 1 mm EGTA, 5 mm sodium pyrophosphate, 50 mm sodium fluoride and protease inhibitor cocktail (Roche). Proteins from mouse heart and brain tissues were extracted using RIPA buffer (Thermo Fisher Scientific) supplemented with protease and phosphatase inhibitors (Thermo Fisher Scientific). Cell lysates were clarified by centrifugation at 14000 *g* for 15 min at 4 °C (for MEFs) or at 17000 x *g* for 15 min at 4 °C (for mouse tissues). Supernatant was collected and stored at −80 °C. Protein concentration in each sample was quantified using the bicinchoninic acid assay kit (Thermo Fisher Scientific). Protein extracted from MEFs was reduced and denatured in 5 X sample buffer containing 4 mm Tris(2‐carboxyethyl)phosphine (TCEP) followed by heating at 90 °C for 5 min. Protein extracted from mouse tissues was reduced and denatured in 4 X sample buffer (Thermo Scientific, NP0007) containing 167 mm dithiothreitol by heating at 70 °C for 10 min. Protein samples were separated by 4–20% SDS/PAGE (BioRad Cat#4561094) for MEF proteins or by 4–12% SDS/PAGE (Thermo Fisher Scientific) for mouse tissue proteins, and electrophoresed at 120 V for 60 min. Gel was transferred onto a polyvinylidene difluoride membrane (GE Healthcare Life Sciences, Marlborough, MA, USA) at 70 V (for MEFs) or 20 V (for mouse tissues) for 90 min at 4 °C V, and subsequently blocked with skim milk (for MEFs) or Intercept Tris‐buffered saline blocking buffer (LiCOR Biosciences, Lincoln, NE, USA) (for mouse tissues) for 1 h at room temperature. Membrane was then probed with Drp1‐specific mouse monoclonal antibody (1.0 μg·mL^−1^, clone 4E11B11, Cell Signalling Technology, Danvers, MA, USA), phospho‐Drp1 (Ser616) antibody (1 in 1000, #3455; Cell Signalling Technology), phospho‐Drp1 (Ser637) antibody (1 in 1000, clone D3A4, #6319; Cell Signaling Technology), or alpha‐tubulin monoclonal antibody (0.36 μg·mL^−1^, clone DM1A; Cell Signalling Technology) at 4 °C overnight. Following successive washes with TBS‐T, membrane was probed with secondary antibody [IRDye 800CW Goat anti‐mouse (0.05 μg·mL^−1^; LiCOR Biosciences) and Goat anti‐rabbit AlexaFluor680, (0.2 μg·mL^−1^, Thermo Fisher Scientific)] at room temperature for 1 h. After washing, membrane was scanned using the Odyssey CLx scanner (LiCOR Biosciences). Band intensities from western blots were quantified by densitometry using ImageJ.

### Mitochondrial morphology analysis

Mitochondrial morphology was assessed using Hsp60 immunostaining on MEFs cultured in 8‐well chamber slides (4000 cells per well, Thermo Fisher Scientific) precoated with 0.1% (w/v) gelatin [39]. Fixed MEFs were stained with Anti‐Hsp60 (2 μg·mL^−1^, rabbit polyclonal, ab46798, Abcam, lot#1071079‐1) overnight at 4 °C followed by Alexa Fluor‐488 goat anti‐rabbit secondary antibody (10 μg·mL^−1^, Invitrogen) and DAPI (1 mg·mL^−1^) for 1 h at room temperature. Coverslips were mounted using fluorescence mounting medium (Dako, Glostrup, Denmark). Images were acquired using an Olympus BX61 fluorescence microscope at 600× magnification. For each replicate, images were captured from 20 random fields of view.

Mitochondrial morphology was evaluated using both manual visual classification and automated AI‐based image analysis. Manual scoring classified cells into two dominant mitochondrial phenotypes, networked or tubular, providing an intuitive, cell‐level overview of mitochondrial organisation. Networked mitochondria were defined as long, highly interconnected, branched structures forming reticular networks spanning the cytoplasm, indicative of fusion‐dominant states. Tubular mitochondria were defined as a mixture of short tubules and small, spherical mitochondria, characterised by discontinuous structures with minimal interconnectivity. Between 194 and 621 cells were analysed per replicate. The combined percentage of cells classified as networked or tubular totalled 100%.

Automated image analysis was used to quantify mitochondrial morphology with high resolution and minimal bias, capturing features of size, shape, network connectivity and structural integrity. Image processing and analysis were performed using FIJI, with mitochondria segmented by adaptive thresholding using the Mitochondrial Analyzer plugin [40] (rolling radius = 2.00 μm, sigma radius = 1.1, block size = 2.15 μm, C‐value = 4, outlier radius = 1.1, median‐based adaptive thresholding). Segmented mitochondria were analysed using the Analyze Particles and Skeletonize functions to extract a focused set of morphological metrics. Mitochondrial size was quantified by area, reflecting overall mitochondrial content. Network connectivity was quantified using interconnectivity as a direct measure of network continuity, together with total branch number and junctions to capture network complexity. Shape was assessed using aspect ratio (major/minor axis) and circularity to distinguish elongated, tubular mitochondria from fragmented structures. Structural integrity was evaluated using solidity to differentiate continuous from fragmented mitochondrial structures. Between 43 and 126 cells were analysed per replicate (between 268 and 483 cells per condition). To enable comparison across metrics with differing units and dynamic ranges, condition‐level mean values for each parameter were z‐score standardised across conditions.

### Recombinant His_6_‐Drp1 expression and purification

cDNAs encoding human Drp1 isoform 1 (UniProt ID: O00429‐1), isoform 2 (UniProt ID: O00429‐3), isoform 3 (UniProt ID: O00429‐4), isoform 4 (UniProt ID: O00429‐2), isoform 5 (UniProt ID: O00429‐6), isoform 6 (UniProt ID: O00429‐8), and isoform 8 (UniProt ID: O00429‐9) were synthesised by General Biosystems with N‐terminal His_6_‐tags and cloned into the pET‐21b(+) vector using *NdeI/NotI* restriction sites. All constructs were sequence‐verified and transformed into *Escherichia coli* Rosetta (DE3) cells (Novagen, Merck Millipore, Burlington, MA, USA). For recombinant protein expression, transformed cells were cultured in 2 L of 2× yeast extract–tryptone medium supplemented with 100 μg·mL^−1^ ampicillin at 37 °C, shaking at 120 rpm, until reaching an optical density of *A*_600_ ~ 1.0. Protein expression was induced with 0.5 mm β‐D‐1‐thiogalactopyranoside, followed by incubation at 16 °C for ~ 16 h. Cells were harvested by centrifugation at 3019 ***g*** at 4 °C for 20 min.

Cell pellets were resuspended in ice‐cold lysis buffer. For all isoforms except isoform 3, the lysis buffer contained 50 mm HEPES (pH 7.55), 400 mm NaCl, 5 mm MgCl_2_, 40 mm imidazole, 2.5 mm β‐mercaptoethanol (BME), 10 μm leupeptin, 100 μm (4‐(2‐aminoethyl)benzenesulfonyl fluoride) (AEBSF), and 1 mm benzamidine hydrochloride. For isoform 3, the buffer contained 50 mm HEPES (pH 7.8), 400 mm NaCl, 5 mm MgCl_2_, 40 mm imidazole, 10 μm leupeptin, 100 μm AEBSF and 1 mm benzamidine hydrochloride. Cells were lysed with a pre‐chilled EmulsiFlex‐C5 homogenizer (Avestin, Ottawa, ON, Canada) and clarified by centrifugation at 16 500 ***g*** at 4 °C for 30 min.

Supernatants were loaded onto Nickel Sepharose Fast Flow 6 resin (GE Healthcare, Chicago, IL, USA) for affinity purification. For all isoforms except isoform 3, proteins were eluted in 50 mm HEPES (pH 7.55), 400 mm NaCl, 5 mm MgCl_2_, 400 mm imidazole and 2.5 mm BME. For isoform 3, elution buffer contained 50 mm HEPES (pH 7.8), 400 mm NaCl, 5 mm MgCl_2_, 500 mm imidazole, and freshly added 5 mm BME. Eluted protein were subjected to size‐exclusion chromatography on a HiLoad 16/600 Superdex 200 pg column (GE Healthcare) equilibrated in storage buffer. For all isoforms except isoform 3, storage buffer contained 20 mm HEPES (pH 7.5), 300 mm NaCl, 2.5 mm MgCl_2_, and 2.5 mm TCEP. For isoform 3, storage buffer contained 20 mm HEPES (pH 7.5), 300 mm NaCl, 2.5 mm MgCl_2_, and freshly added 5 mm BME. Purified Drp1 proteins were aliquoted, flash‐frozen in liquid nitrogen, and stored at −80 °C.

### Mass photometry

Mass photometry measurements of Drp1 were performed on the Two MP system (Refyn, Waltham, MA, USA). The instrument was calibrated using bovine serum albumin and thyroglobulin. Measurements were performed in a total volume of 40 μL per well. For each experiment, 10 μL of MP buffer (20 mm HEPES (pH 7.5), 300 mm NaCl, 2.5 mm MgCl_2_ and 2.5 mm TCEP) were added to clean wells to calibrate the focal height. Freshly thawed Drp1 protein was diluted in MP buffer, and 10 μL of the protein solution was added to the wells to achieve a final concentration of 200 nm, with and without 2 mm GMPPCP. Each measurement was recorded for 120 s, and at least three independent replicates were performed for each Drp1 isoform. Molecular masses of detected species between 65 and 1200 kDa were determined using the calibration curve.

### Surface plasmon resonance (SPR)

All SPR experiments were performed using a Biacore T200 instrument (GE Healthcare Life Sciences) at 25 °C in HBS‐EP+ buffer (20 mm HEPES, pH 7.4, 150 mm NaCl, 50 μm EDTA, and 0.005% Tween‐20). Drp1 protein was tethered to a NiHC1500 SPR sensor chip (Xantec, Düsseldorf, Germany) via His‐tag capture in SPR buffer. Incremental injections of the protein were performed to achieve a surface density of approximately 8000 RU (flow rate 10 μL·min^−1^), corresponding to a calculated maximum analyte binding capacity (*R*_max_) of ~ 45 RU. The immobilised protein surface was allowed to stabilise for at least 1 h in HBS‐EP+ buffer prior to analyte injection. GDP (Merck, Darmstadt, Germany) was dissolved in HBS‐EP+ buffer to prepare a 100 mm stock solution and subsequently diluted to desired concentrations. The compound was injected across the chip surface at 30 μL·min^−1^ for 60 s, followed by a 400 s dissociation phase. A threefold serial dilution series was used, starting from a maximum concentration of 120 μm (10‐point dilution series). Data were analysed using the Biacore T200 Evaluation Software version 1.

### Drp1 GTPase activity assay

Malachite green reagent was prepared by mixing 0.0812% (w/v) malachite green (M‐9636, Sigma‐Aldrich) in Milli‐Q water, 2.32% (w/v) polyvinyl alcohol (P8136‐250, Sigma‐Aldrich) in Milli‐Q water (dissolved with heating), 5.72% (w/v) ammonium molybdate (A7302, Sigma‐Aldrich) in 6 m HCl, and Milli‐Q water at a volume ratio of 2 : 1 : 1 : 2. The mixture was incubated at room temperature in the dark for approximately 2 h before use. Recombinant human Drp1 (0.5 μm for all isoforms, except for isoform 2, which was used at 0.25 μm) was incubated with 0.5 mm GTP (R0461, Thermo Fisher Scientific) in assay buffer containing 50 mm HEPES (pH 7.5), 2 mm MgCl_2_, and 1 mm dithiothreitol at 37 °C in the dark for 60 min, in a final volume of 50 μL in a 96‐well microtiter plate. The concentration for each isoform was empirically determined to remain within the assay's linear range. Reactions were terminated by addition of 12.5 μL of 0.5 m EDTA (pH 8.0), followed by 50 μL malachite green reagent. Subsequently, 5 μL of 34% sodium citrate was added to each well to prevent nonenzymatic hydrolysis of ATP from contributing to background signal [41]. Plates were incubated at room temperature in the dark for 30 min. Inorganic phosphate release from GTP hydrolysis was quantified by measuring absorbance at 650 nm using a CLARIOstar Plus plate reader (BMG Labtech, Ortenberg, Germany). Negative controls included reactions lacking either Drp1 or GTP. All assays were performed in triplicate, and each experiment was repeated independently at least three times.

### Statistics

All values are expressed as mean ± standard error of the mean (SEM). Significance of the differences was evaluated using unpaired Student's *t*‐test or one‐way ANOVA followed by multiple comparison *post hoc* analysis where appropriate. *P* ≤ 0.05 was considered statistically significant.

## Conflict of interest

The authors declare no conflict of interest.

## Author contributions

SYL conceived the project, designed and performed the experiments, and analysed and interpreted data. FY, JSJ, NXLL, AMK, JQT, MLM, AAR and LZ planned and performed experiments and analysed data. AAR, CL, JGL, SB‐S, DAO, AP, MD, KL and SL provided materials and methodologic input. NMD supervised the data analysis. FY, JH, NMD, JSO and SYL provided interpretative review of study findings. All authors participated in manuscript preparation and approved the final version of the manuscript.

## Supporting information


**Fig. S1.** Read length distribution.
**Fig. S2**. Unique Molecular Identifier (UMI) saturation analysis.
**Fig. S3**. 5′–3′ transcript and exonic coverage profiles of *DNM1L*/Dnm1l.
**Fig. S4**. IGV view of *DNM1L/Dnm1L* sequence coverage.
**Fig. S5**. Expression of *Dnm1l* transcript variants in mouse tissues.
**Fig. S6**. Expression of Drp1 and phosphorylated Drp1 in heart and brain tissues of db/+ and db/db mice.
**Fig. S7**. Quantitative analysis of mitochondrial morphology.

## Data Availability

The long‐read Nanopore RNA sequencing data generated in this study have been deposited in the Gene Expression Omnibus under accession number GSE308731 (https://www.ncbi.nlm.nih.gov/geo/query/acc.cgi?acc=GSE308731). GTEx bulk long‐read RNA sequencing data were downloaded from dbGAP under accession number phs000424.v9 (https://www.omicsdi.org/dataset/dbgap/phs000424).
