# Supplementary material for: Long‐read sequencing‐based atlas of tissue‐specific expression of DNM1L transcript variants
Source: FEBS J. 2026 Apr 29;293(20):6208–29. doi: 10.1111/febs.70568 (PMC13669652; doi:10.1111/febs.70568)
Supplement: Supplementary file 1 [file FEBS-293-6208-s001.pdf]

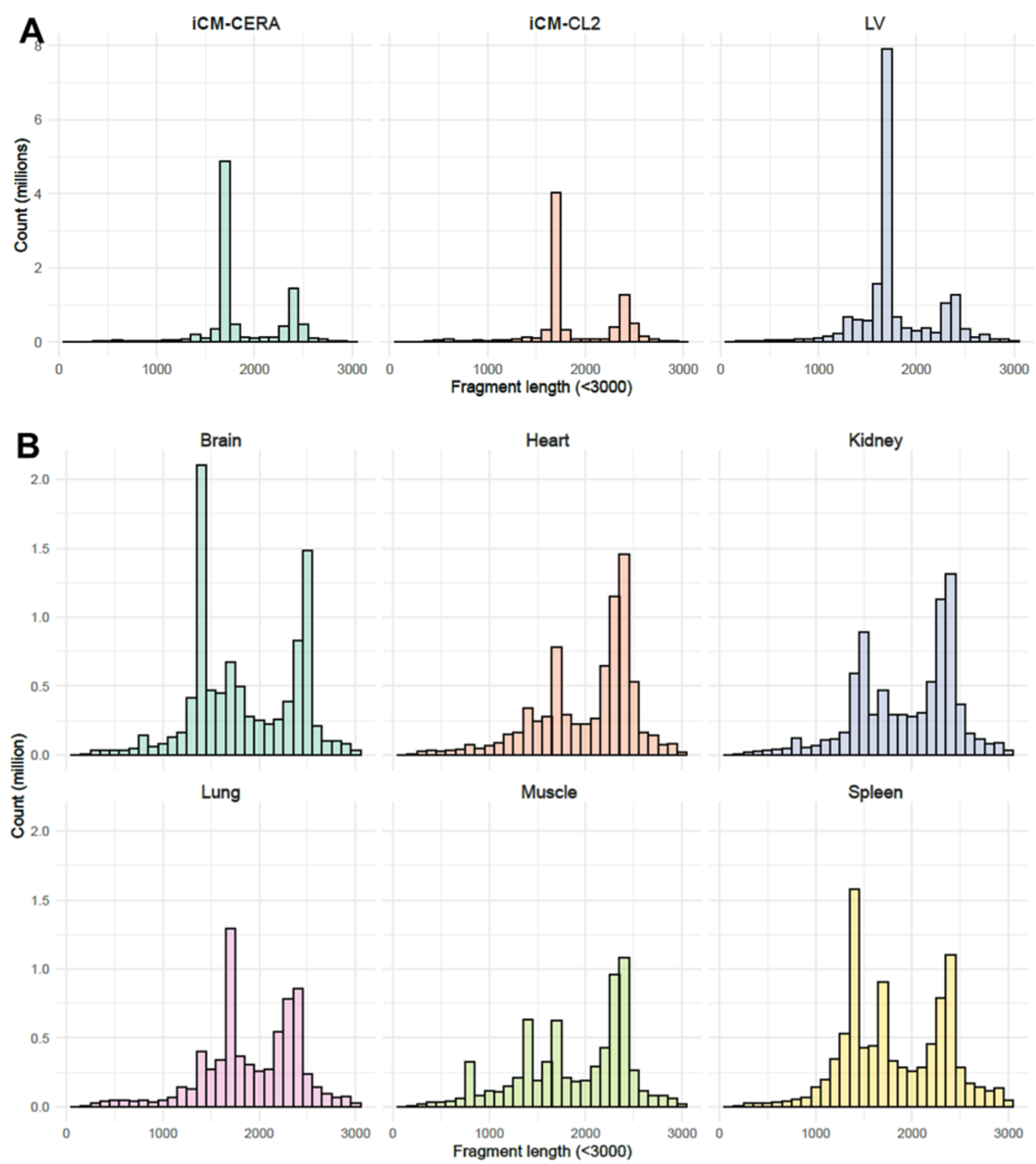

Supplementary Figure 1. Read length distribution. Read length distributions across biological human **(A)** and mouse **(B)** samples.

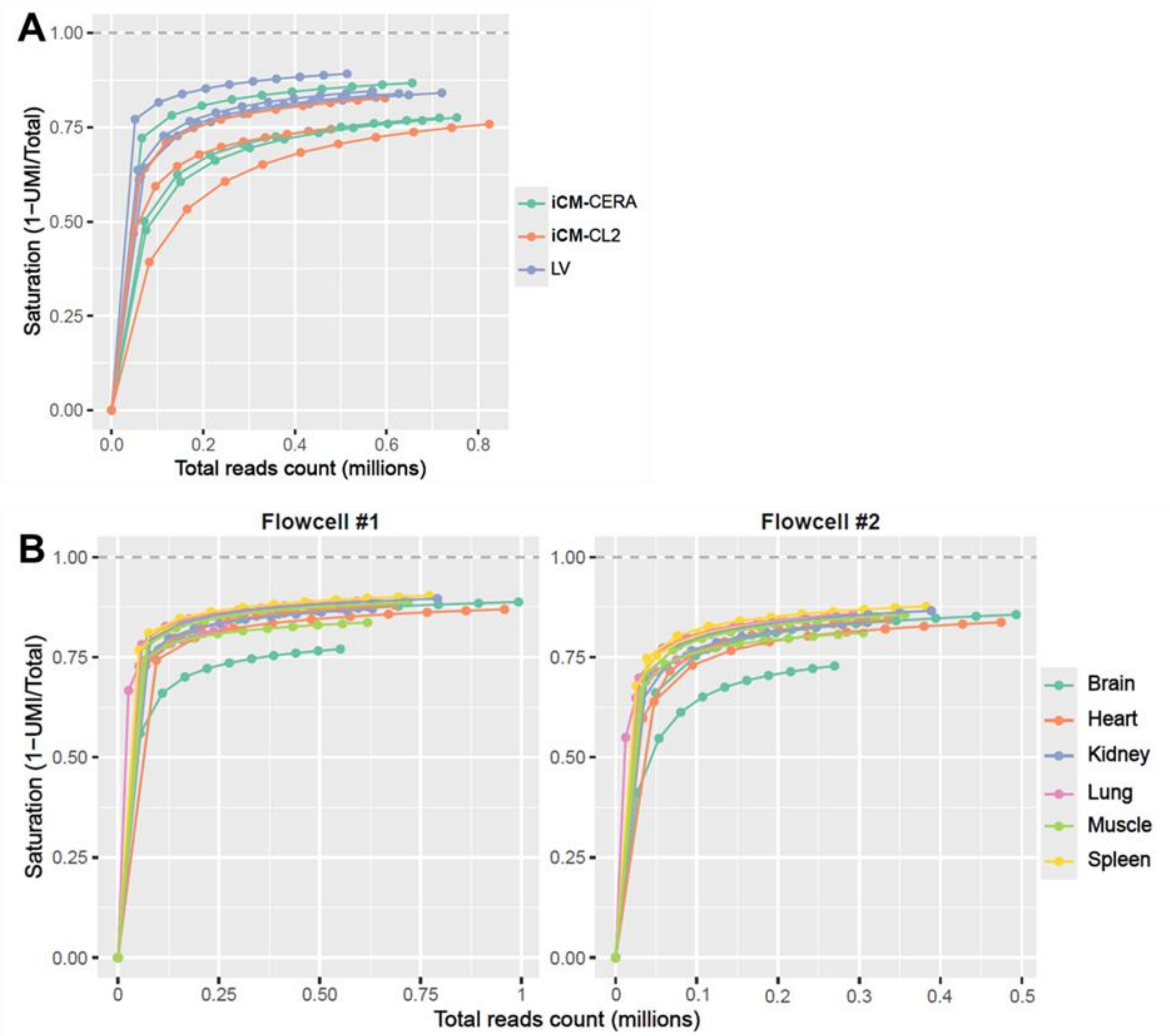

Supplementary Figure 2. Unique Molecular Identifier (UMI) saturation analysis. Unique Molecular Identifier (UMI) saturation curve of human **(A)** and mouse **(B)** samples. For each sample, sequencing data was subsampled to defined percentages of the total mapped reads. UMI deduplication was then performed on each subsampled dataset to determine the number of unique UMI reads detected at each depth. Mouse data from two flowcells are shown separately.

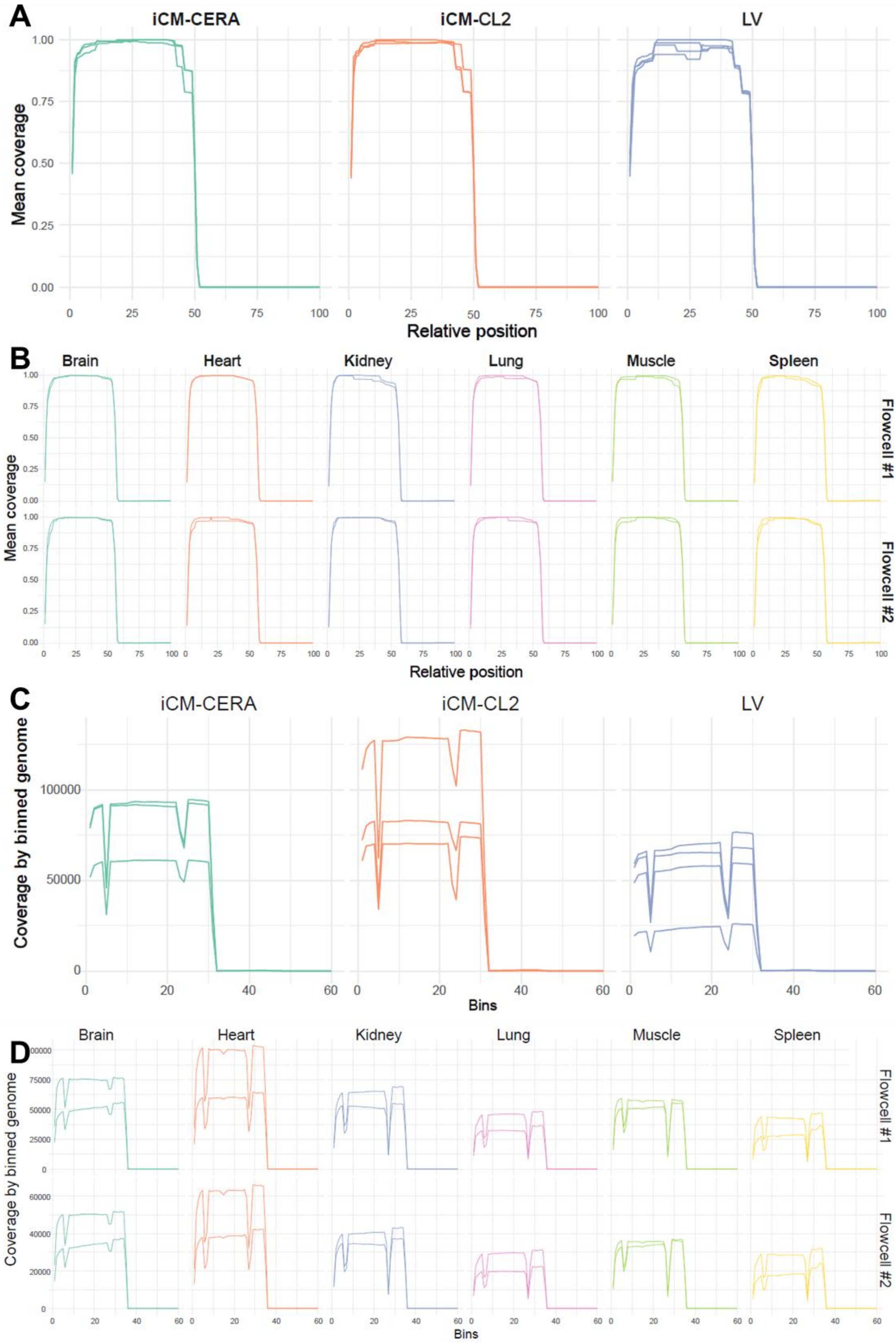
A
iCM-CERA
iCM-CL2
LV
Mean coverage
Relative position
B
Brain
Heart
Kidney
Lung
Muscle
Spleen
Flowcell #1
Flowcell #2
Mean coverage
Relative position
C
iCM-CERA
iCM-CL2
LV
Coverage by binned genome
Bins
D
Brain
Heart
Kidney
Lung
Muscle
Spleen
Flowcell #1
Flowcell #2
Coverage by binned genome
Bins

Supplementary Figure 3. 5′–3′ transcript and exonic coverage profiles of *DNM1L*/Dnm1l. (A-B) 5'–3' transcript coverage across binned position in human **(A)** and mouse **(B)** samples. Mouse data from two flowcells are shown separately. For each transcript, coverage was calculated as the read depth within each bin normalized by the total reads aligned to that transcript. Coverage profiles of all *DNM1L* transcripts were averaged and plotted on the y-axis. The sharp drop in coverage corresponds to the location of the designed probes (~ 2200 bp from the 5' end, near exon 21, representing ~50% of the transcript length). The downstream ~50% that is underrepresented corresponds primarily to the 3′ UTR. **(C-D)** Exonic coverage by binned position in human **(C)** and mouse **(D)** samples. Mouse data from two flowcells are shown separately. The coverage shown in y axis is the number of reads at each bin of concatenated human *DNM1L* 21 exon sequences. The drop of the line aligns with the location of exon 3 and 16, 17.

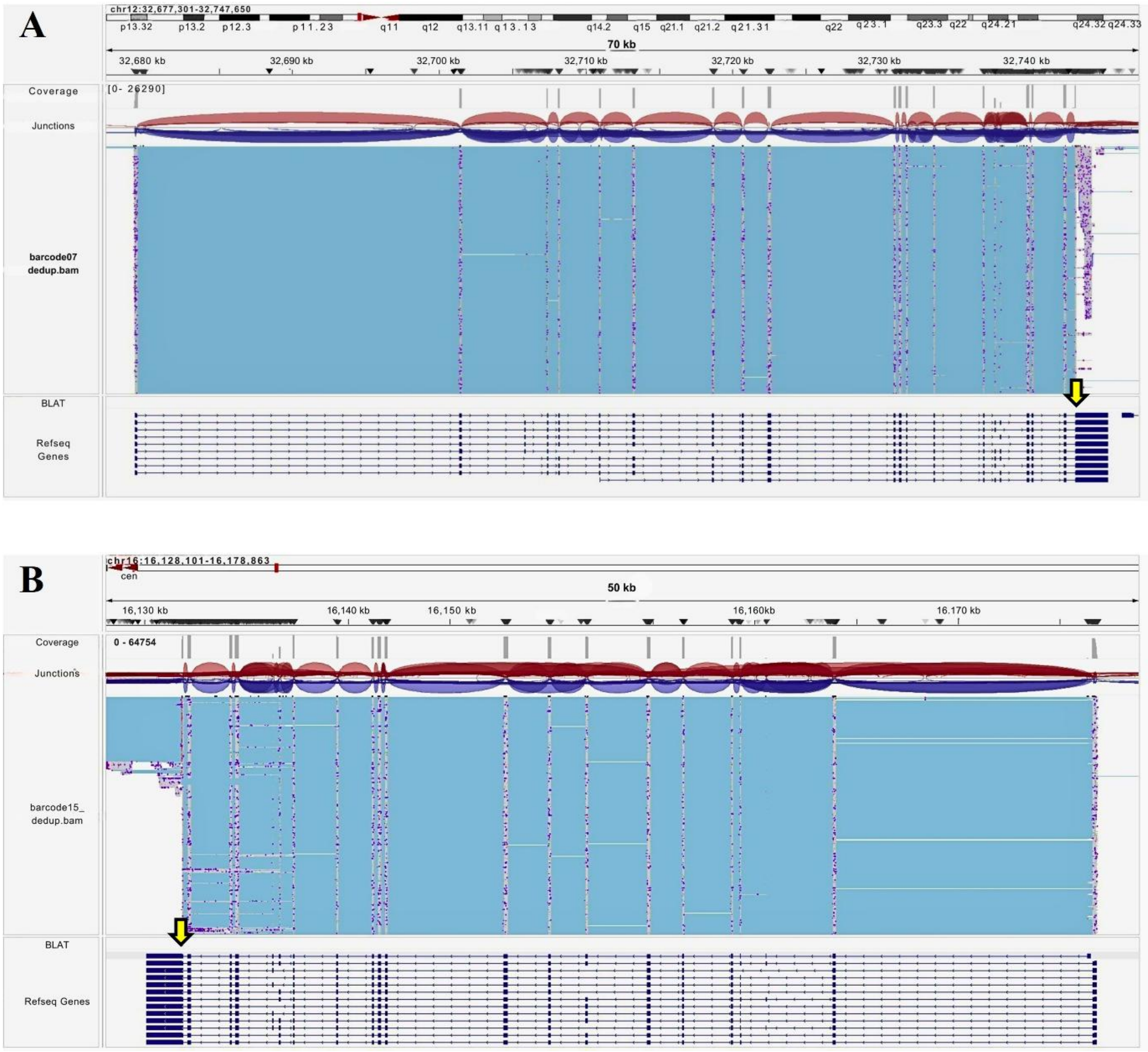

Supplementary Figure 4: IGV view of *DNM1L/Dnm1L* sequence coverage. IGV view of the sequence coverage of the *DNM1L/Dnm1L* in human left ventricle **(A)** and mouse heart **(B)** samples. Yellow arrows indicate the binding position of the primer designed to target exon 21 for amplification.

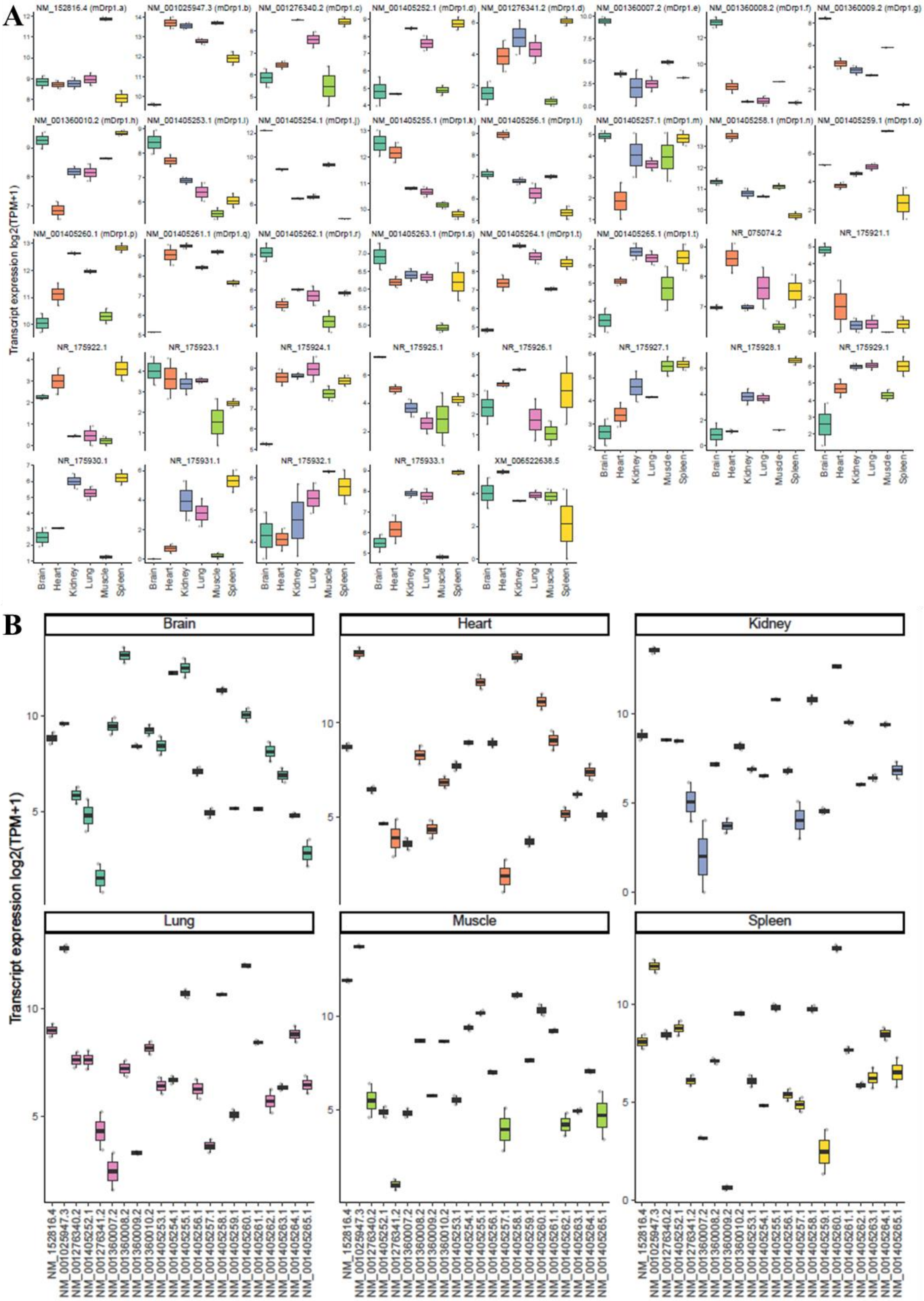

Supplementary Figure 5: Expression of *Dnm1l* transcript variants in mouse tissues. (A) Boxplots showing expression levels [log2(TPM+1)] of individual *Dnm1l* transcripts across tissue samples. **(B)** Boxplots showing expression levels [log2(TPM+1)] of individual *Dnm1l* transcripts in different tissue types.

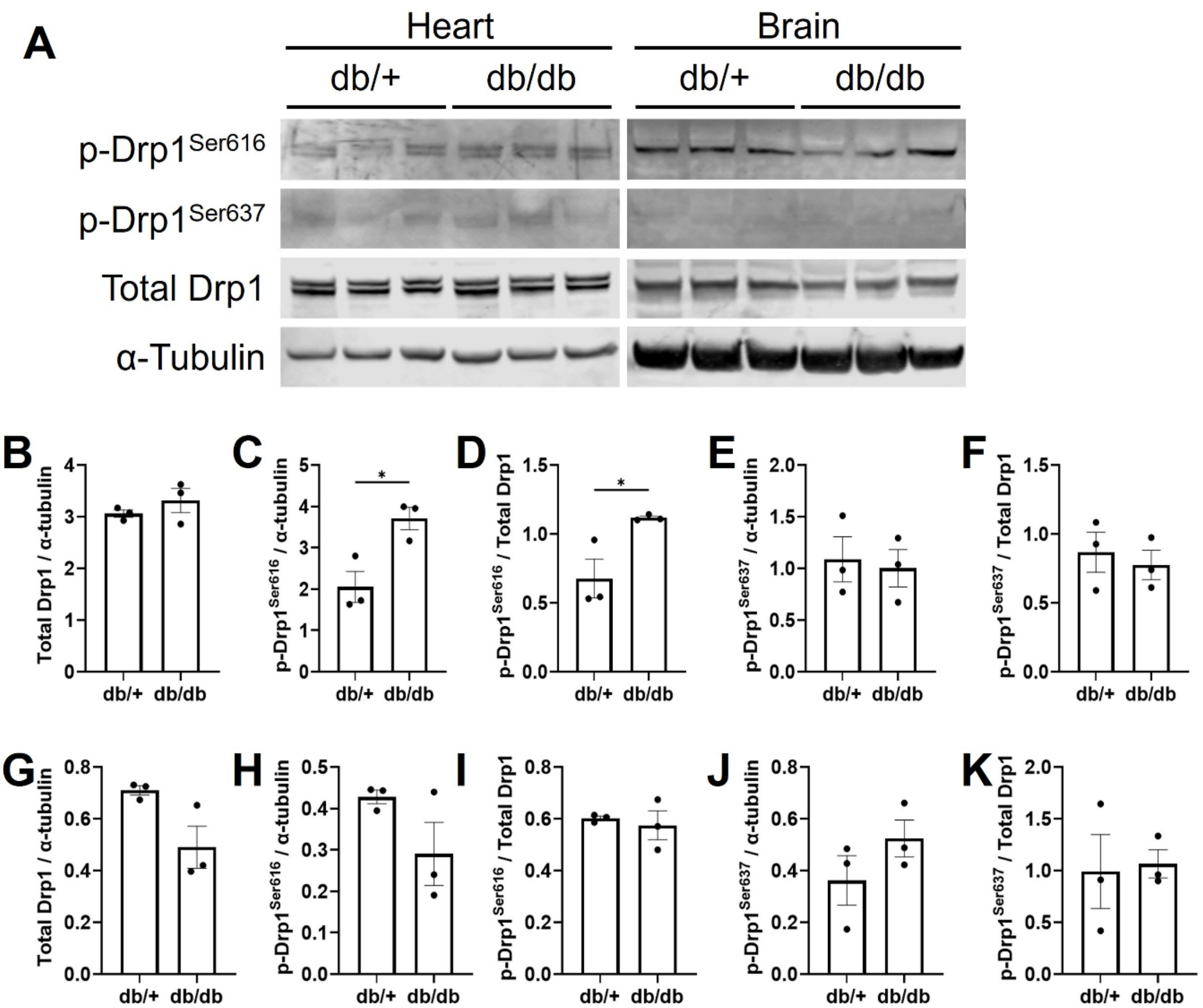

Supplementary Figure 6: Expression of **Drp1 and phosphorylated Drp1 in heart and brain tissues of db/+ and db/db mice. (A)** Representative immunoblots and semi-quantitative analyses of total Drp1, phosphorylated Drp1 at Ser616 (p-Drp1^{Ser616}), phosphorylated Drp1 at Ser637 (p-Drp1^{Ser637}), and α-tubulin in heart and brain tissues from db/+ and db/db mice. **(B-F)** Semi-quantitative analysis in heart tissue showing total Drp1 **(B)**, p-Drp1^{Ser616} normalised to α-tubulin **(C)** or total Drp1 **(D)**, and p-Drp1^{Ser637} normalised to α-tubulin **(E)** or total Drp1 **(F)** in heart tissues of db/+ and db/db mice. **(G-K)** Semi-quantitative analysis in brain tissue showing total Drp1 **(G)**, p-Drp1^{Ser616} normalised to α-tubulin **(H)** or total Drp1 **(I)**, and p-Drp1^{Ser637} normalised to α-tubulin **(J)** or total Drp1 **(K)** in brain tissues of db/+ and db/db mice. N=3 mice per group. Data are presented as mean ± SEM. *$P < 0.05$ as determined by unpaired t-test.

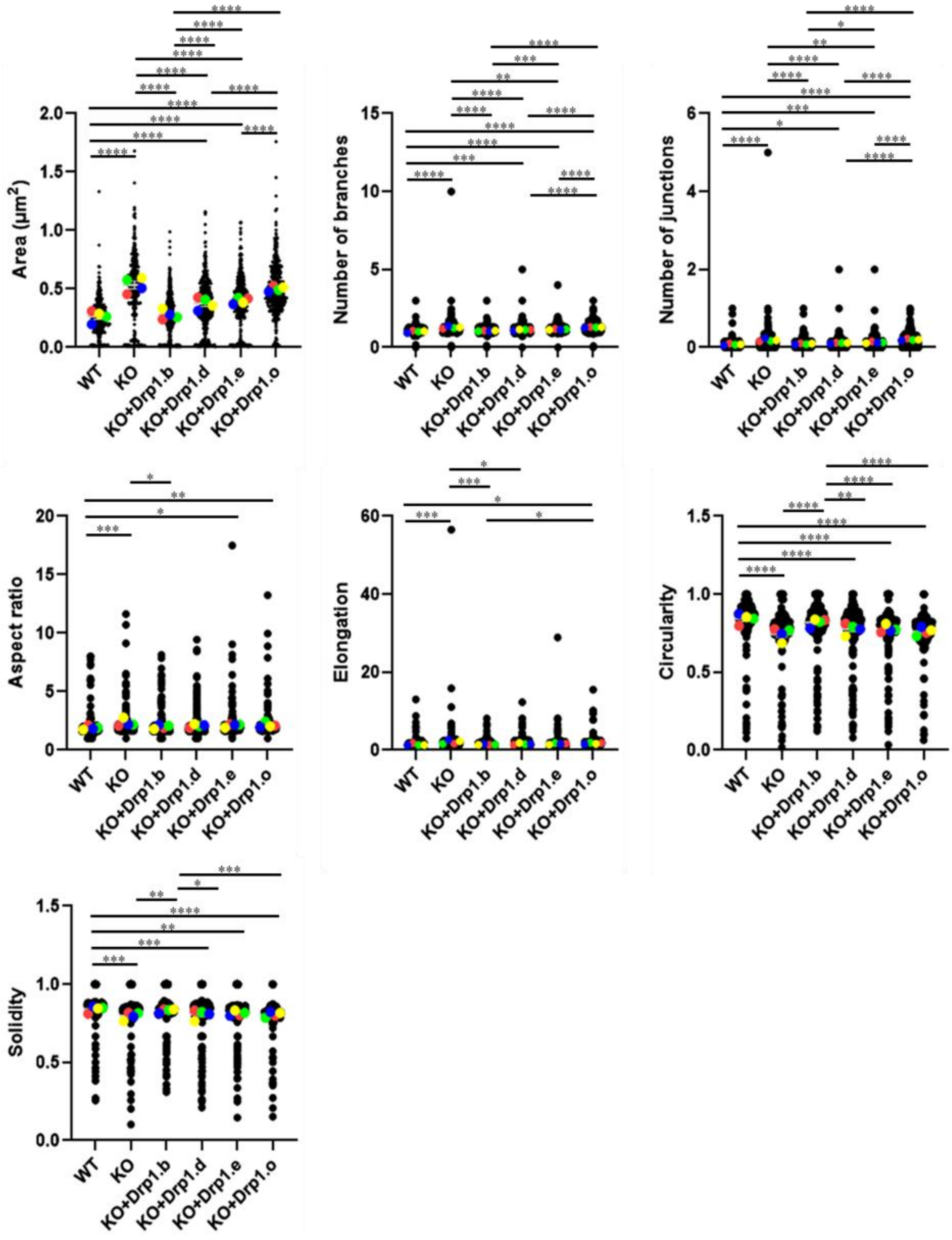

Supplementary Figure 7: Quantitative analysis of mitochondrial morphology. Automated image analysis was performed in FIJI, with mitochondrial morphology in individual cells quantified following segmentation by adaptive thresholding using the Mitochondrial Analyzer plugin. The analysis measured mitochondrial area, branch count, junction count, aspect ratio, elongation, circularity, and solidity. N=4 replicates. Data are expressed as mean ± SEM. *$p < 0.05$, **$p < 0.01$, ***$p < 0.001$, ****$p < 0.0001$ by one-way ANOVA with Bonferroni post hoc test.
